# Genomic and Metabolite Profiling Reveal a Novel *Streptomyces* Strain, QHH-9511, from the Qinghai-Tibet Plateau

**DOI:** 10.1128/spectrum.02764-22

**Published:** 2023-01-09

**Authors:** Xi-Long Feng, Rui-Qi Zhang, Da-Cheng Wang, Wei-Ge Dong, Zhen-Xin Wang, Yi-Jie Zhai, Wen-Bo Han, Xia Yin, Junmian Tian, Jing Wei, Jin-Ming Gao, Jianzhao Qi

**Affiliations:** a Shaanxi Key Laboratory of Natural Products & Chemical Biology, College of Chemistry & Pharmacy, Northwest A&F University, Yangling, Shaanxi, China; b College of Biology Pharmacy & Food Engineering, Shangluo University, Shangluo, Shaanxi, China; c Qinba Mountains of Bio-Resource Collaborative Innovation Center of Southern Shaanxi Province, Hanzhong, Shaanxi, China; Huazhong Agricultural University

**Keywords:** novel *Streptomyces*, phylogenomic analysis, aromatic polyketides, type II PKS, biosynthetic potential

## Abstract

The prevalence of superbugs, represented by methicillin-resistant Staphylococcus aureus (MRSA), has become a serious clinical and public safety concern with rising incidence in hospitals. Polyketides with diverse chemical structures harbor many antimicrobial activities, including those of rifampin and rapamycin against MRSA. *Streptomyces* sp. QHH-9511 was isolated from a niche habitat in the Qinghai-Tibet Plateau and used to produce antibacterial metabolites. Herein, an integrated approach combining genome mining and metabolic analysis were employed to decipher the chemical origin of the antibacterial components with pigmented properties in strain QHH-9511, a novel *Streptomyces* species from a lichen symbiont on the Qinghai-Tibet Plateau. Genomic phylogeny assembled at the chromosome level revealed its unique evolutionary state. Further genome mining uncovered 36 candidate gene clusters, most of which were uncharacterized. Meanwhile, based on liquid chromatography coupled to diode array detection mass spectrometry, a series of granaticins, BSMs, chromones, phaeochromycins, and related molecules were discovered by using the Global Natural Product Social molecular networking platform. Subsequently, several pigment compounds were isolated and identified by high-resolution mass spectrometry and/or nuclear magnetic resonance, among which the structure-activity relationships of seven aromatic polyketides showed that the fused lactone ring of the C-2 carboxyl group could increase antibacterial activity. Genetic experiments indicated that all seven aromatic polyketides are a series of metabolic shunts produced by a single type II polyketide synthase (PKS) cluster. Comparative genomic analysis of granaticin producers showed that the granaticin gene cluster is widely distributed. This study provides an efficient method to combine genome mining and metabolic profiling techniques to uncover bioactive metabolites derived from specific habitats, while deepening our understanding of aromatic polyketide biosynthesis.

**IMPORTANCE** Undescribed microorganisms from special habitats are being screened for anti-superbug drug molecules. In a project to screen actinomycetes for anti-MRSA activity, we isolated a *Streptomyces* strain from Qinghai Lake lichens. The phylogeny based on the genome assembled at the chromosome level revealed this strain's unique evolutionary state. The chemical origins of the antibacterial components with pigment properties in strain QHH-9511 were determined using an integrated approach combining genome mining and metabolic analysis. Further genome mining uncovered 36 secondary metabolite gene clusters, the majority of which were previously unknown. A series of aromatic compounds were discovered using molecular network analysis, separation, and extraction. Genetic experiments revealed that all seven aromatic polyketides are a series of metabolic shunts produced by a single cluster of type II PKSs. This study describes a method for identifying novel *Streptomyces* from specific habitats by combining genome mining with metabolic profiling techniques.

## INTRODUCTION

In recent years, the crisis of antibiotic resistance, represented by methicillin-resistant Staphylococcus aureus (MRSA), has triggered a radical revolution in natural product discovery strategies (1). In the search for compounds capable of fighting life-threatening infections caused by multidrug-resistant pathogens, as well as meeting previously unmet therapeutic needs, screening activities to discover new compounds have shifted from the traditional strong preference for soil-dwelling actinomycetes to environments beyond the land, such as the deep ocean and the polar regions (2, 3). Actinomycetes from unconventional environments are emerging as promising sources of novel bioactive compounds (4), such as insect-associated *Streptomyces*, which inhibit drug-resistant pathogens more effectively than soil-derived *Streptomyces* (5).

*Streptomyces*, a representative group of Actinomycetes, have extraordinary metabolic potential, as revealed by genome sequencing (6), although the biosynthetic potential of most of these species has not yet been fully demonstrated. Genome mining can dereplicate discovery efforts and hence accelerate bioactivity-guided isolation by discounting the strains missing the biosynthetic gene clusters of interest. Furthermore, genome mining can estimate biosynthetic potential and aid in chemical structure elucidation (7, 8). Genome-guided studies investigating *Streptomyces* have been recently reported and reviewed (9, 10), and new informatic approaches are rapidly expanding the accessibility of this powerful approach (11). The continuous improvement of high-throughput sequencing technology and metagenomics research methods has led to a fundamental change in microbial genomic research from single genome characterization to large-scale pangenomic analysis (12, 13). Bioinformatics-based genome mining coupled with metabolic profiling is emerging as a powerful tool for targeted (14) or untargeted (15, 16) natural product discovery.

Polyketides, especially concerning *Streptomyces*, are common metabolites of actinomycetes, and as a class of natural products, they harbor vast diversity in chemical structure and biological activity, including a long history of success as antimicrobial agents (8, 17, 18). The chemical diversity and commercial value of polyketides have motivated tremendous efforts to understand their biosynthesis, since total chemical synthesis often remains challenging and is increasingly being supplanted by more sustainable biocatalytic processes (19). The biosynthesis of aromatic polyketides is typically performed by type II polyketide synthases (T2PKSs), which consist of a heterodimer keto-synthase (KS) with KS_α_ and KS_β_ (chain length factor) domains catalyzing consecutive Claisen condensations between enzyme-bound thioesters of the growing polyketide chain and extender units typically originating from malonyl-coenzyme A (malonyl-CoA) (20) and loaded onto an acyl carrier protein. Keto-reductases, cyclases, and aromatases serve to fashion an aromatic scaffold from the polyketide backbone. Tailoring enzymes, such as oxygenases and methyl- or glycosyltransferases, further modify the aromatic scaffold to furnish a final biosynthetic product (21, 22). The benzoisochromanequinones (BIQs), a family of antibiotics including alnumycin (23), qinimycin (24), medermycins (25), and actinorhodin (26) as well as granaticins (27), are representative type II polyketides from *Streptomyces*. Formation of the core naphtho-[2,3-*c*]-pyran-5,10-dione ring structure is catalyzed by type II PKSs, with structural diversity arising from the action of post-PKS tailoring enzymes (28). The complex pi-conjugation of the core ring system leads to different visible absorption spectra for different BIQs.

Here, we report a new strain, *Streptomyces* sp. QHH-9511, isolated from the brown lichen symbionts on the Qinghai-Tibet Plateau, whose unique metabolic profile caught our attention. The genome was assembled at the chromosome level using a combination of Illumina Hiseq with PacBio RSII sequencing technologies, while phylogenomic and average nucleotide identity analyses based on chromosomal sequences confirmed its unique evolutionary status. Genome mining using bioinformatics identified 36 candidate biosynthetic gene clusters (BGCs), with large parts being undescribed BGCs. BIG-SCAPE-based gene cluster family analysis further revealed the uniqueness of these BGCs. On the other hand, molecular network analysis of metabolites and the isolation and structural identification of monomeric compounds demonstrated the ability to produce secondary metabolites of this strain. The structure-activity investigation of aromatic compounds demonstrated the importance of specific functional groups. Inactivation of *orf26*-*28* in cluster 20 suggested that seven compounds come from the same gene cluster. Comparative genomic analysis of granaticin producers revealed a varied distribution of granaticin BGCs. This study combines genome and metabolite profiling to characterize novel lichen-derived *Streptomyces* on the Tibetan Plateau and add new insights into aromatic polyketide biosynthesis.

## RESULTS

### Genome characteristics of *Streptomyces* sp. QHH-9511.

As part of a screening aimed at identifying extracts of natural products with MRSA inhibition activity, we identified the candidate *Streptomyces* sp. QHH-9511, a strain isolated from yellow lichen on a rocky surface on the west coast of Qinghai Lake (see Fig. S1A and B in the supplemental material). Specifically, 3 days after inoculating mycelium on a Gause’s agar plate, a blue-violet pigment diffused through the medium (Fig. S1C). An agar plug bioassay revealed anti-MRSA properties (Fig. S1D), and its absorption spectrum was clearly pH dependent (Fig. S1E). These findings prompted a whole-genome sequencing to dissect the genetic basis of biosynthesis of these compounds in *Streptomyces* sp. QHH-9511.

**FIG 1:**
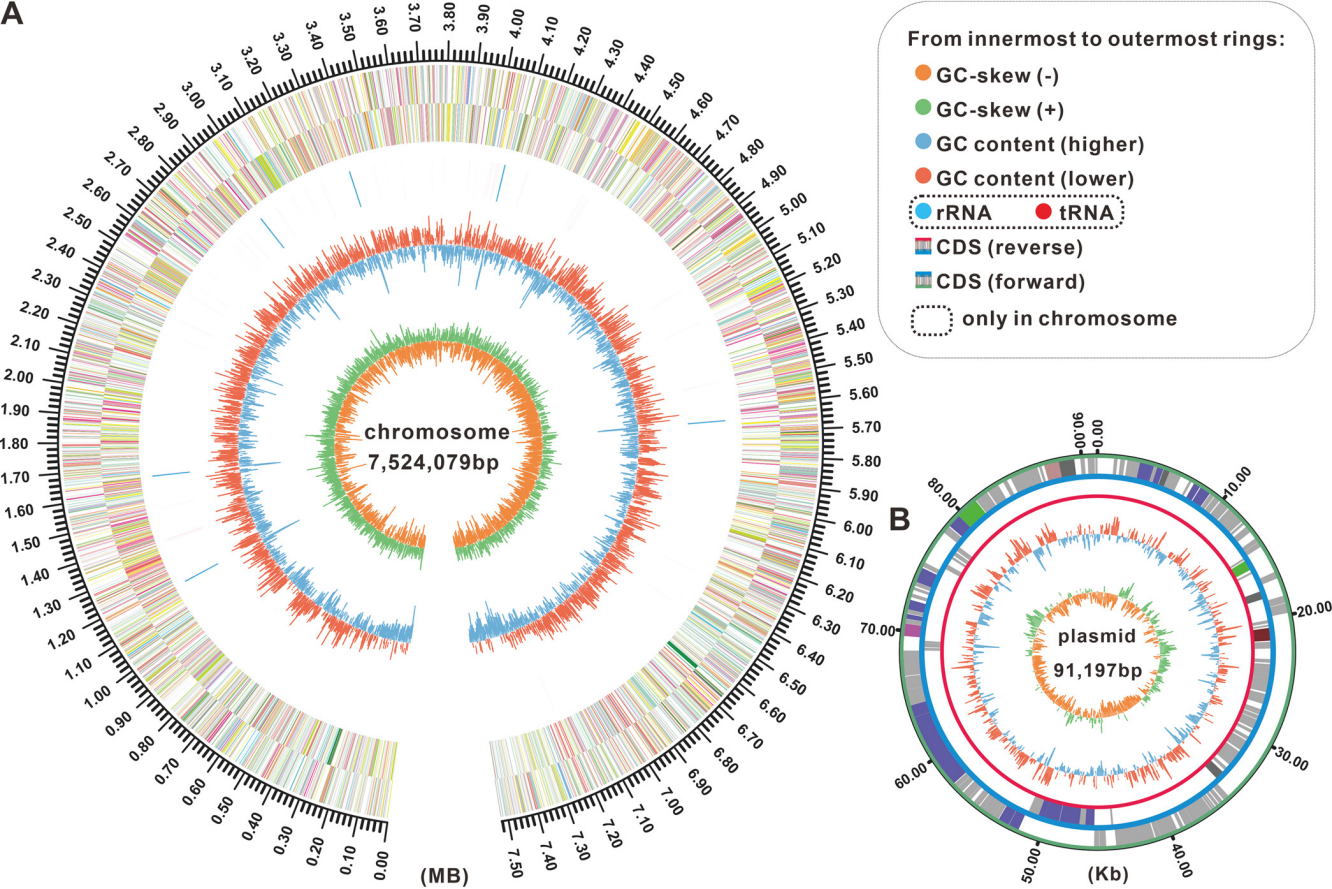
The chromosomal genome and plasmid maps of *Streptomyces* sp. QHH-9511. (A) Visualization of the chromosomal genome of *Streptomyces* sp. QHH-9511. The outermost circle of the circle diagram is a marker for genome size. The second and third circles are the coding DNA sequence (CDS) on the positive and negative strands, with different colors indicating the functional classification of the different COGs of the CDS. The fourth circle is for rRNA (deep sky blue) and tRNA (pure red). The fifth circle is for GC content, with the outward red part indicating that the GC content of the region is higher than the genome-wide average GC content, with higher peaks indicating a greater difference from the average GC content, and the inward blue part indicating that the GC content of the region is lower than the genome-wide average GC content, with higher peaks indicating a greater difference from the average GC content. The innermost circle is the GC-skew value, which is based on the algorithm G−C/G+C. (B) The plasmid map is missing the fourth circle (from outside to inside); the rest of the circle map information is consistent with the chromosomal genomic map.

The genome of *Streptomyces* sp. QHH-9511 consists of a linear chromosome of 7,524,079 bp with 71.34% GC content (Fig. 1A) and a circular plasmid of 91,197 bp with 69.42% GC content (Fig. 1B). A total of 7,555 genes in the chromosomal DNA sequence and 128 genes in the plasmid DNA sequence could be functionally annotated in the NCBI non-redundant database. There are 64 tRNA, 18 rRNA operons, and 3 noncoding RNAs, as well as 495 pseudogenes predicted in the chromosome (Table S1). Tandem repeats are ubiquitous features of prokaryotic genomes (29), and the cumulative total length of the predicted 418 random repeats was 55,230 bp, accounting for 0.83% of the whole genome in this strain. Gene length distribution frequency statistics found that most genes were less than 2 kb in length (Fig. S2).

Functional gene analysis using GO, COG (Fig. S3), and KEGG (Fig. S4) categories revealed that the number of genes involved in metabolic processes was higher than the number of function-related genes annotated in GO (2,416) and KEGG (1,742), which implies that the producer possesses powerful metabolic capacities. *Streptomyces* species are thought to acquire biosynthetic gene clusters through horizontal gene transfer, thereby enabling them to quickly start producing various secondary metabolites (30). There are 22 genomic island regions in the chromosome (Table S2). These intriguing genomic features evoked our fascination with the evolutionary taxonomic status and species affinities of this strain.

### Taxonomic status of *Streptomyces* sp. QHH-9511.

**FIG 2:**
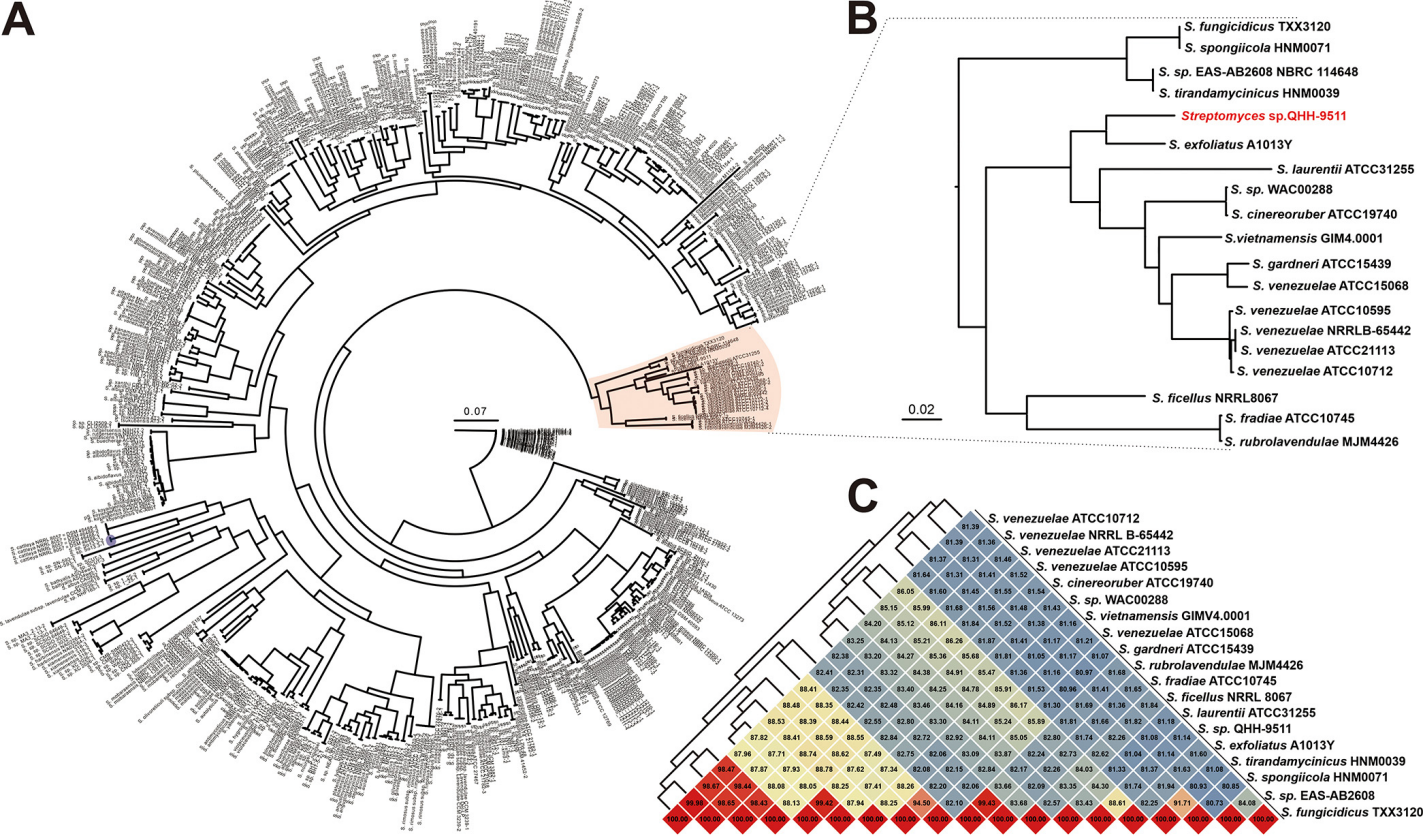
Comparative genomic analyses based on core genes and unique genes. (A and B) Phylogenomic tree analysis of strain QHH-9511 and related species. (C) ANI analysis of the clade within strain QHH-9511. The heatmap presenting ANI values was implemented through the ComplexHeatmap package in R.

A genome-wide comparative analysis of strain QHH-9511 and the currently available 623 *Streptomyces* species with complete genome assemblies revealed these strains share 156 single-copy orthologous genes. Phylogenomic analyses revealed that strain QHH-9511 is in a distinct clade (Fig. 2A), which consists of 19 *Streptomyces* strains forming three subgroups. Among them, strain QHH-9511 and Streptomyces exfoliatus A013Y (31) are in the smallest cluster, indicating that strain QHH-9511 has the closest relationship with *S. exfoliatus* A1013Y (Fig. 2B).

Furthermore, fastANI was used to analyze the average nucleotide identity (ANI) of strain QHH-9511 and its closely related strains on the phylogenomic tree. The result revealed that the ANI value of strain QHH-9511 and *S. exfoliatus* A1013Y was 88.61%, while strain QHH-9511 and other strains had ANI values below this value (Fig. 2C). As the ANI value was higher than 95%, which is the level used to distinguish between bacterial species (32), strain QHH-9511 can be considered a new *Streptomyces* species among the currently known strains with whole-genome sequencing available. The novel evolutionary status of QHH-9511 hinted at its novel genomic content and motivated us to analyze the biosynthetic potential of its secondary metabolites.

### Analysis of secondary metabolite BGCs and resistance genes.

**FIG 3:**
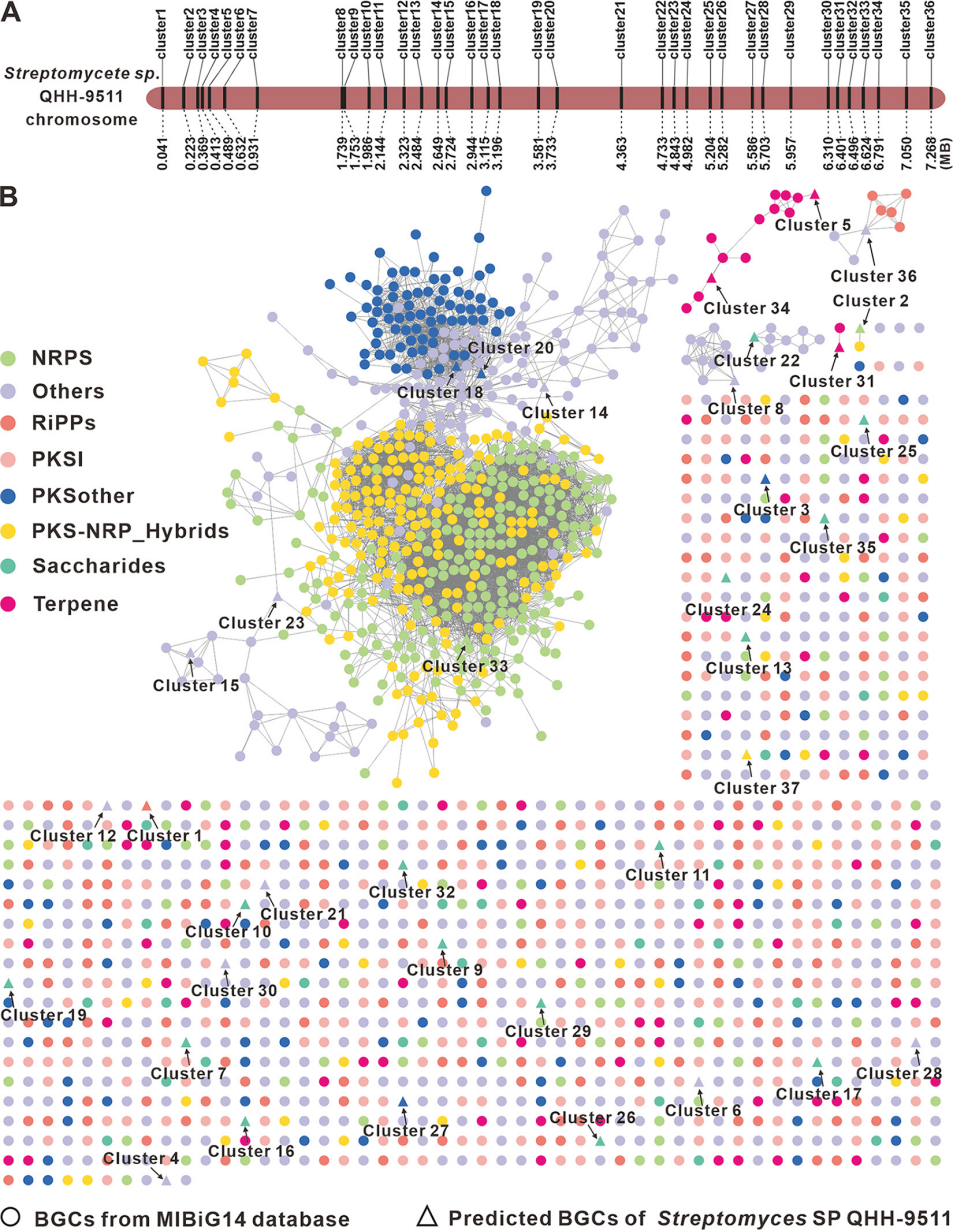
Prediction and analysis of BGCs from strain QHH-9511. (A) The predicted BGC distribution on the chromosome of strain 9511 determined by antiSMASH 6.1. (B) Similarity network of the BGCs was obtained using BiG-SCAPE. Each node represents a BGC, and those with similar Pfam domain metrics are connected by edges. A cutoff 0.80 was used for the analysis, and the final similarity network was visualized using Cytoscape 3.9.1.

The genome of strain QHH-9511 has a considerable number of candidate BGCs for various metabolites based on *in silico* analysis, with up to 36 putative BGCs (clusters 1 to 36) (Fig. 3A, Table 1) for secondary metabolite unevenly distributed on the 7.52-Mb linear chromosome, as determined by antiSMASH 6.1 and manual inspection. Among these BGCs, five BGCs with 100% similarity were predicted to be responsible for alkylresorcinol (cluster 3), geosmin (cluster 5), ectoine (cluster 8), desferrioxamine B (cluster 15), and lanthipeptide SapB (cluster 36) synthesis (Fig. S5), respectively. In fact, these five BGCs are highly conserved in *Streptomyces* and are present in almost all sequenced *Streptomyces* genomes, since they were first discovered in the genome of Streptomyces coelicolor A3(2) (6). In terms of product type, a total of 18 gene clusters are predicted to be saccharide clusters, 16 of which are associated only with saccharide. In addition, the BGCs encoded five polyketides (including alkylresorcinol), three nonribosomal peptide synthases (NRPSs), three terpenes (including geosmin), two siderophores (including desferrioxamin), and a ribosomally synthesized and posttranslationally modified peptide (RiPP; SapB), with six other compounds predicted. Moreover, a BGC (cluster 37) encoding a T3PKS was predicted on the plasmid.

**TABLE 1:** Putative BGCs responsible for secondary metabolites in strain QHH-9511^*a*^

Cluster	Type	From	To	The most similar known cluster	Type	Similarity
Cluster 1	Thiopeptide, LAP	41,014	73,109	Rustmicin	T1PKS	10%
Cluster 2	NRP-like	223,464	272,882	RP-1776	PKS+NRPS	4%
Cluster 3	T3PKS	369,257	408,542	Alkylresorcinol	PKS	100%
Cluster 4	NRPS-like	412,514	453,801	Ficellomycin	NRPS	3%
Cluster 5	Terpene	488,979	510,344	Geosmin	Terpene	100%
Cluster 6	Indole	631,988	653,286	Terfestatins	Other	23%
Cluster 7	Saccharide	930,787	961,645	Spiroindimicins	Other	6%
Cluster 8	Ectoine	1,739,486	1,749,884	Ectoine	Other	100%
Cluster 9	Saccharide	1,752,906	1,788,706	SF2575	T2PKS	6%
Cluster 10	Saccharide	1,986,380	2,010,125	—	—	—
Cluster 11	Saccharide	2,143,976	2,176,677	Acarviostatin l03	Other	33%
Cluster 12	Fatty acid	2,323,396	2,343,972	Asukamycin	T2PKS	3%
Cluster 13	Saccharide	2,484,142	2,507,803	—	—	—
Cluster 14	Butyrolactone	2,649,169	2,658,735	Lactonamycin	PKS	8%
Cluster 15	Siderophore	2,724,349	2,735,300	Desferrioxamine B	Other	100%
Cluster 16	Saccharide	2,943,607	2,986,285	—	—	—
Cluster 17	Saccharide	3,114,622	3,132,144	—	—	—
Cluster 18	T2PKS, butyrolactone	3,196,272	3,267,701	Prejadomycin	T2PKS	35%
Cluster 19	Saccharide	3,580,756	3,614,715	—	—	—
Cluster 20	T2PKS	3,733,235	3,805,735	Granaticin	T2PKS	97%
Cluster 21	Halogenated	4,362,997	4,384,280	Caniferolides	T1PKS	3%
Cluster 22	Melanin	4,732,520	4,797,322	Istamycin	Other	8%
Cluster 23	Ladderane, NRPS	4,842,639	4,894,497	S56-p1	NRPS	43%
Cluster 24	Saccharide	4,982,219	5,005,140	—	—	—
Cluster 25	Saccharide	5,203,825	5,223,871	—	—	—
Cluster 26	PKS-like	5,281,781	5,375,426	BD-12	NRPS	14%
Cluster 27	T3PKS	5,585,817	5,626,950	Flaviolin	Other	50%
Cluster 28	Siderophore	5,703,020	5,717,593	Ficellomycin	NRPS	3%
Cluster 29	Saccharide	5,956,687	5,988,380	—	—	—
Cluster 30	Fatty acid	6,309,806	6,330,753	—	—	—
Cluster 31	Terpene	6,400,943	6,426,745	Hopene	Terpene	69%
Cluster 32	Saccharide	6,495,979	6,543,997	Glycopeptidolipid	NRPS	5%
Cluster 33	NRPS	6,624,104	6,690,623	Gobichelins	NRPS	55%
Cluster 34	Terpene	6,790,978	6,812,015	—	—	—
Cluster 35	Saccharide	7,050,117	7,071,533	—	—	—
Cluster 36	Lanthipeptide	7,268,275	7,291,028	SapB	Lanthipeptide	100%

a Secondary metabolite types detected by antiSMASH: T1PKS, type I PKS cluster; T2PKS, type II PKS cluster; T3PKS, type III PKS cluster; NRPS, nonribosomal peptide synthetase cluster; other cluster containing a secondary metabolite, related protein that did not fit into any other category. The similarity is the percentage of homologous genes in the query cluster that are present in the hit cluster of the antiSMASH database. —, not available.

Biosynthetic gene similarity clustering and prospecting engine (BiG-SCAPE), a bioinformatic software package, was subsequently used to analyze these 36 predicted BGCs (Fig. 3B) in detail, and we found that only 13 BGCs established gene cluster family (GCF) similarity networks with BGCs derived from MIBiG14, of which 6 BGCs (including cluster 15) were located on a complex GCF network consisting of known BGCs responsible for PKS, PKS-NRPS, NRPS, and other BGCs. Cluster 15 and the four BGCs responsible for desferrioxamine B formed a subcluster, and these gene clusters were highly similar (Fig. S5A). Although cluster 3 showed 100% similarity to specific known clusters analyzed by antiSMASH (Fig. S5B), it did not form a GCF network in BiG-SCAPE analysis. Clusters 5 and 34 are in a GCF network composed entirely of terpenes. Although cluster 5 shows 100% similarity to the known specific BGCs from antiSMASH (Fig. S5C), it shows low similarity to most of its neighboring BGCs in the GCF network (Fig. S5D). Cluster 8 and cluster 22 are respectively in two small GCF networks composed of other cluster 8 and its intracluster members exhibiting high conservation (Fig. S5E). Cluster 31 is associated with the BGC (BGC0000631) for hopene (Fig. S5F), with a similarity of 63%. Cluster 2, a putative BGC for NRP, displays an association with a BGC for PKS-NRPS. Cluster 36 is located at the connection point of a GCF network composed of RiPPs and others, and cluster 36 is highly similar to these RiPP BGCs (Fig. S5G).

Antibiotic-resistant target seeker (ARTS) 2.0 was used to further understand the genome, and the characterization of the BGCs was predicted by antiSMASH. A total of 42 resistance models were identified, including genes coding for ATP-binding cassette antibiotic efflux pumps, biotin-dependent carboxylases, DNA topoisomerase, pentapeptide repeats, carboxyl transferase, glyceraldehyde 3-phosphate dehydrogenase, etc. Analysis of BGC proximity revealed that 20 BGCs harbored either neighboring putative core genes (clusters 9 and 17), known resistance genes (clusters 1, 2, 3, 5, 6, 8, 10, 12, 18, 20, and 21), both genes (clusters 4, 11, 13, 14, 15, 16, and 19), or unknown resistance genes (Table S3).

The antiSMASH prediction results showed that 30 of the 37 predicted BGCs were less than 50% similar to known BGCs, indicating that strain QHH-9511 contains many undescribed BGCs responsible for novel compounds. The high number of antibiotic resistance genes implies that the strain is equipped with complex defense systems and some of the BGCs are involved in resistance mechanisms. Therefore, additional studies are needed to further characterize these clusters, as well as to identify and characterize the compounds produced and match them with the corresponding BGCs.

### Molecular network analysis of secondary metabolites.

**FIG 4:**
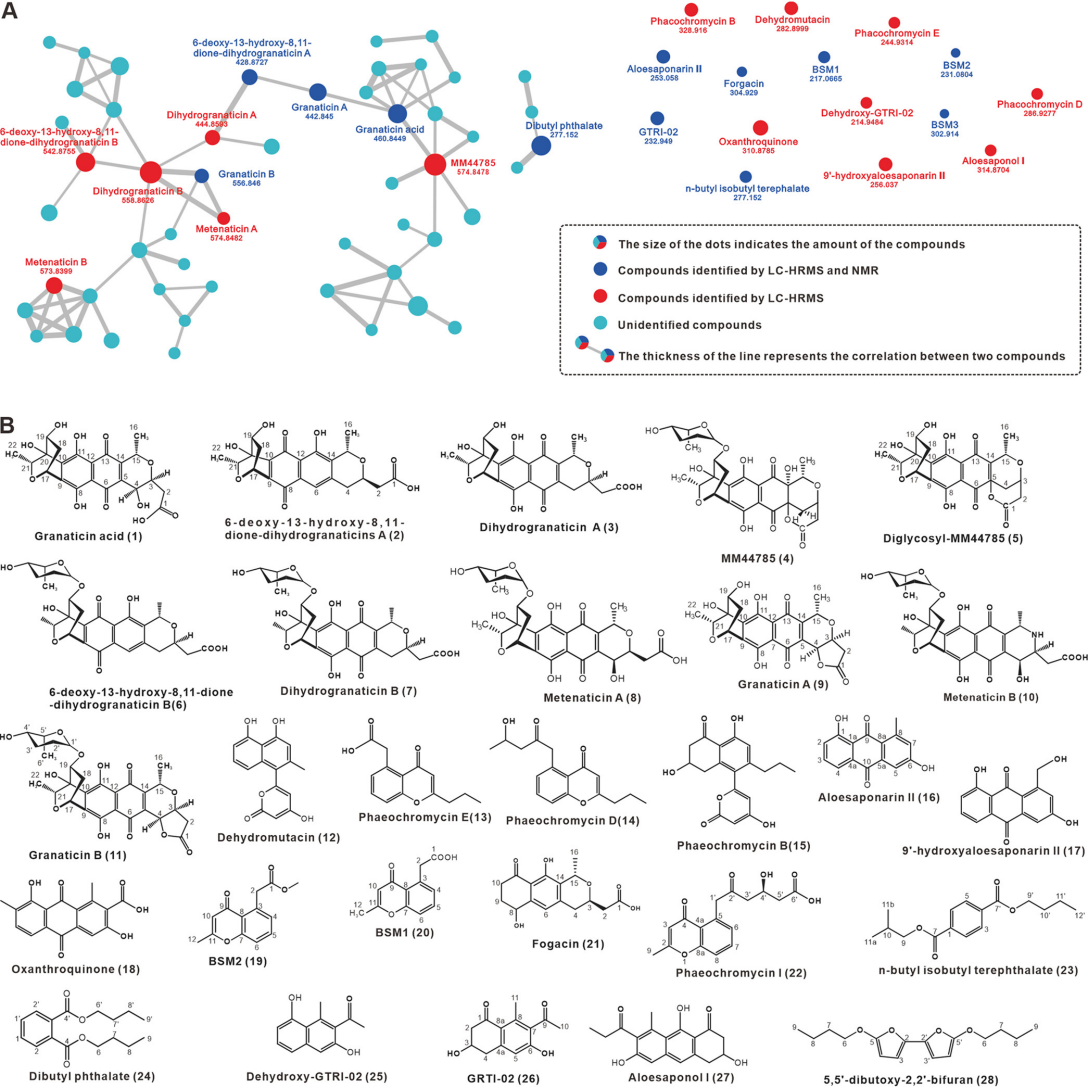
Molecular network analysis of the metabolites of strain QHH-9511 (A) and identified compounds (B). The presentation of molecular networks was achieved through Cytoscape 3.9.1.

Concurrent with genome sequencing and analysis, strain QHH-9511 was used in solid and liquid fermentation to produce secondary metabolites. High-performance liquid chromatography coupld with diode array detection (HPLC-DAD) fingerprint analysis showed that the metabolites of solid fermentation were more abundant than those of liquid fermentation (Fig. S6). The detection and analysis of concentrated crude samples of ethyl acetate extracts from solid fermentation of strain QHH-9511 by GNPS indicated that the metabolites contained large numbers of unknown small molecules, and at least six pigment molecules were present among the identified molecules (Fig. 4A, Table 2, and Fig. S7). Almost all the pigment secondary metabolites identified on the GNPS molecular network were aromatic polyketides, such as BIQ compounds **4**, **8**, and **9**, klavuzon compound **16**, anthraquinone compound **18**, and chromone compound **20**. In addition, benzoic acid derivative **27** was detected (Fig. 4B). The fact that these identified compounds possess complex conjugation systems that enable them to have specific UV and visible light absorptions explains the high production of purplish metabolites by strain QHH-9511 in fermentation cultures. However, it was not known which pigment molecules conferred the antimicrobiological ability of the producer. Thus, these secondary metabolites were further isolated and purified based on their color characteristics for structural determination by nuclear magnetic resonance (NMR) spectral analyses and for the biological activity assay.

**TABLE 2:** Isolated secondary metabolites detected by HR-LC-MS in strain QHH-9511

Metabolite type	Observed mass peak [M-H]^−^	Relative error (ppm)	Retention time (min)	Assignment (compound no.)	Yield (mg/liter)
BIQs	461.1098	1.52	9.661	Granaticin acid (**1**)	4.35
	429.1173	−4.19	12.347	6-Deoxy-13-hydroxy-8,11-dione-dihydrogranaticin A (**2**)	1.63
	445.1138	−1.35	12.221	Dihydrogranaticin A (**3**)	4.57
	575.1763	−1.56	17.646	MM44785 (**4**)	NA^*a*^
	461.1137	−0.65	13.556	Diglycosyl-MM44785 (**5**)	NA
	543.1861	0.73	16.203	6-Deoxy-13-hydroxy-8,11-dione-dihydrogranaticin B (**6**)	NA
	559.1830	0.72	16.268	Dihydrogranaticin B (**7**)	NA
	575.1785	2.09	13.136	Metenaticin A (**8**)	NA
	443.1006	5.19	18.079	Granaticin A (**9**)	5.63
	574.1942	1.57	19.072	Metenaticin B (**10**)	NA
	557.1648	−2.87	22.613	Granaticin B (**11**)	7.88
Klavuzons	283.0620	1.06	11.374	Dehydromutacin (**12**)	NA
	245.0816	−2.04	10.239	Phaeochromycin E (**13**)	NA
	287.1301	3.83	14.137	Phaeochromycin D (**14**)	NA
	329.1028	−1.82	19.667	Phaeochromycin B (**15**)	NA
Anthraquinones	253.0509	−2.77	28.903	Aloesaponarin II (**16**)	5.63
	269.0463	−1.49	24.476	9′-Hydroxyaloesaponarin II (**17**)	NA
	311.0564	0.96	7.056	Oxanthroquinone (**18**)	NA
Chromones	231.0669	−2.6	8.168	BSM2 (**19**)	4.68
	217.0512	2.76	9.064	BSM1 (**20**)	2.80
	305.1042	3.61	9.789	Fogacin (**21**)	2.33
	303.0868	−0.99	10.389	Phaeochromycin I (**22**)	2.38
Benzoic acid derivatives	277.1461	3.61	45.658	*n*-Butyl isobutyl terephalate (**23**)	4.15
	277.1448	−1.8	47.632	Dibutyl phthalate (**24**)	2.70
Others	215.0705	−6.51	7.954	Dehydroxy-GTRI-02 (**25**)	NA
	233.0822	1.29	11.608	GTRI-02 (**26**)	1.75
	314.0885	4.14	23.484	Aloesaponol I (**27**)	NA
	277.1452	−1.8	40.633	5,5′-Dibutoxy-2,2′-bifuran (**28**)	3.13

a NA, not available.

### Isolation, structural identification, and bioactivity evaluation of pigmental compounds.

The crude extract of ethyl acetate in 4 liters of Gause's medium of the strain 9511 was defatted and separated by normal-phase chromatography, reversed-phase chromatography, and gel chromatography (Sephadex LH-20) to obtain relatively pure monomeric compounds. Next, these compounds were purified by semipreparative HPLC to afford 29 known compounds, which were identified as granaticin acid (**1**), 6-deoxy-13-hydroxy-8,11-dione-dihydrogranaticin A (**2**), dihydrogranaticin A **(3**), MM44785 (**4**), diglycosyl-MM44785 (**5**), 6-deoxy-13-hydroxy-8,11-dione-dihydrogranaticin B (**6**), dihydrogranaticin B (**7**), metenaticin A (**8**), granaticin A (**9**), metenaticin B (**10**), granaticin B (**11**), dehydromutacin (**12**), phaeochromycin E (**13**), phaeochromycin D (**14**), phaeochromycin B (**15**), aloesaponarin II (**16**), 9′-hydroxyaloesaponarin II (**17**), oxanthroquinone (**18**), BSM2 (**19**), BSM1 (**20**), fogacin (**21**), phaeochromycin I (**22**), *n*-butyl isobutyl terephalate (**23**), dibutyl phthalate (**24**), dehydroxy-GTRI-02 (**25**), GTRI-02 (**26**), aloesaponol I (**27**), and 5,5′-dibutoxy-2,2′-bifuran (**28**) on the basis of comparisons of their MS and/or NMR data with literature values (Fig. 4B, Fig. S8 and S9, and Table S4).

**FIG 5:**
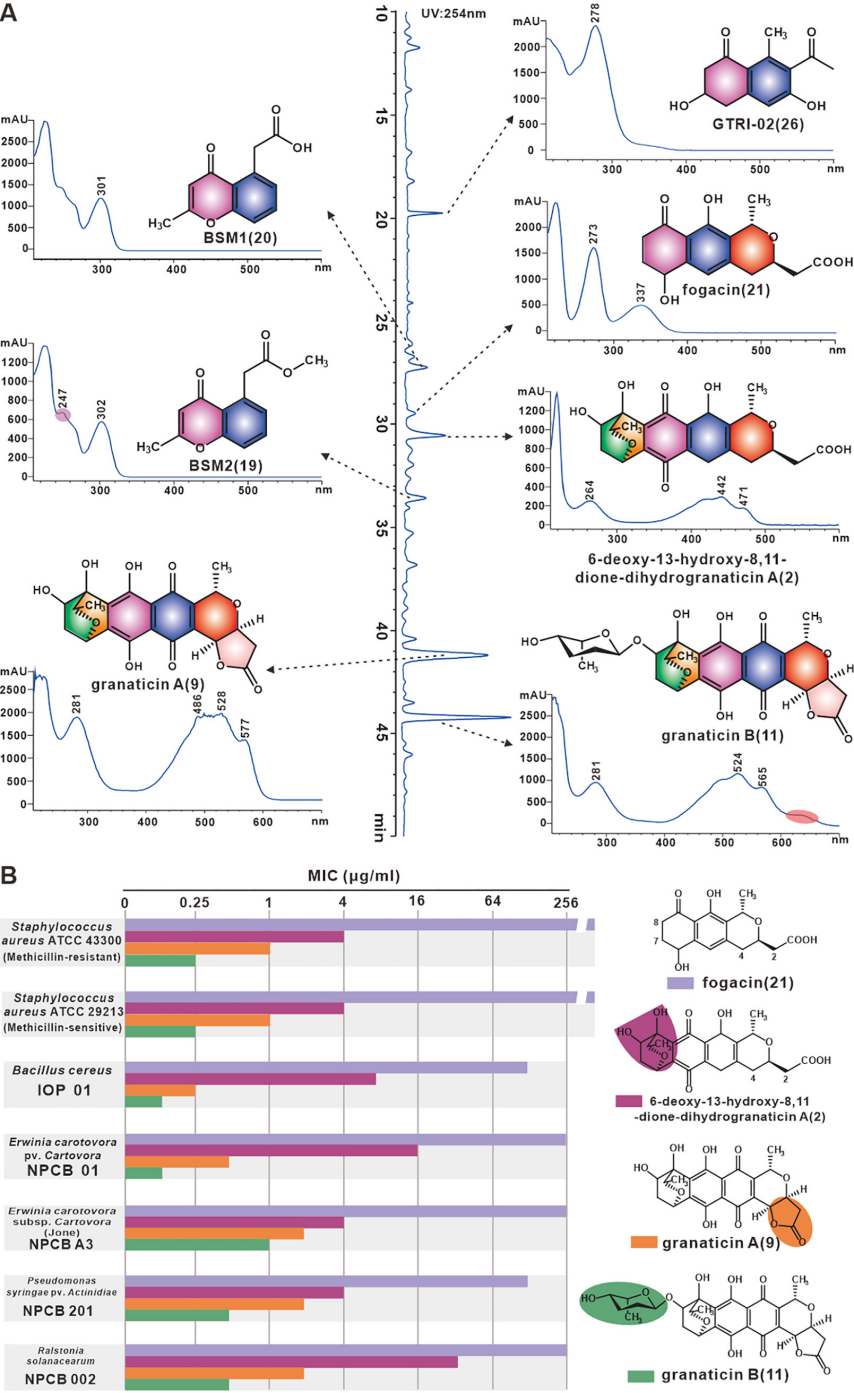
Seven aromatic polyketides and their SARs. (A) The structures, spectral signatures, and retention times of the seven compounds. (B) SARs of compounds **2**, **9**, **11**, and **21**. Structural differences are highlighted in color.

With the monomeric compounds obtained, the retention times and spectral features of seven aromatic polyketide compounds with significant UV-Vis absorption were characterized (Fig. 5A). These compounds were immediately used to determine their antibacterial activity in order to identify the monomeric compounds that conferred antibacterial activity of strain QHH-9511. We next evaluated the MICs of the seven compounds against a series of pathogens, including methicillin-susceptible Staphylococcus aureus ATCC 29231, Bacillus cereus, Erwinia carotovora pv. *carotovora*, Erwinia carotovora subsp. *carotovora* (Jone), Pseudomonas syringae pv. *actinidiae*, and Ralstonia solanacearum. The MIC results corresponded with structural complexity. The more inhibitory compounds, **2**, **9**, and **11**, displayed lower MICs (Table S5), with compound **11** having a MIC of 0.125 μg/mL against both Bacillus cereus and Erwinia carotovora pv. *carotovora*, indicating their antimicrobial potential.

Significantly, the antibacterial results of these compounds also revealed striking structure-activity relationships (SARs) among granaticin analogs. First, the tetracyclic scaffold is clearly essential for antibacterial activity (Fig. 5B), with the presence of the bicyclo-skeleton at C-7 and C-8 resulting in a dramatic activity increase (at least 16-fold MIC reduction for **2** relative to **24**). Second, the γ-lactone linking the C-4 hydroxyl and the C-2 carboxyl may moderately increase antimicrobial activity of compound **9** relative to that of compound **2** (Fig. 5B). Although there are minor differences on other tested bacteria, compound **2** and **9** show 32-fold MIC difference in B. cereus. Third, the rhamnosyl group clearly improves the antibacterial activity of **11**, which only differed from **9** in this respect. Indeed, the MIC values of compound **11** were lower than those of compound **9** in all tested strains (Fig. 5B).

### BGC identification of seven aromatic polyketides.

Compound separation and identification, as well as antimicrobial activity assays, indicated that the strain QHH-9511 has a distinct metabolic profile due to its seven aromatic polyketides. To our knowledge, this is the first time that these seven aromatic polyketides (2, 9, 11, 19–21, 26) have been reported from the same strain, although previous work had speculated that they all originated from a type II PKS (33–37).

**FIG 6:**
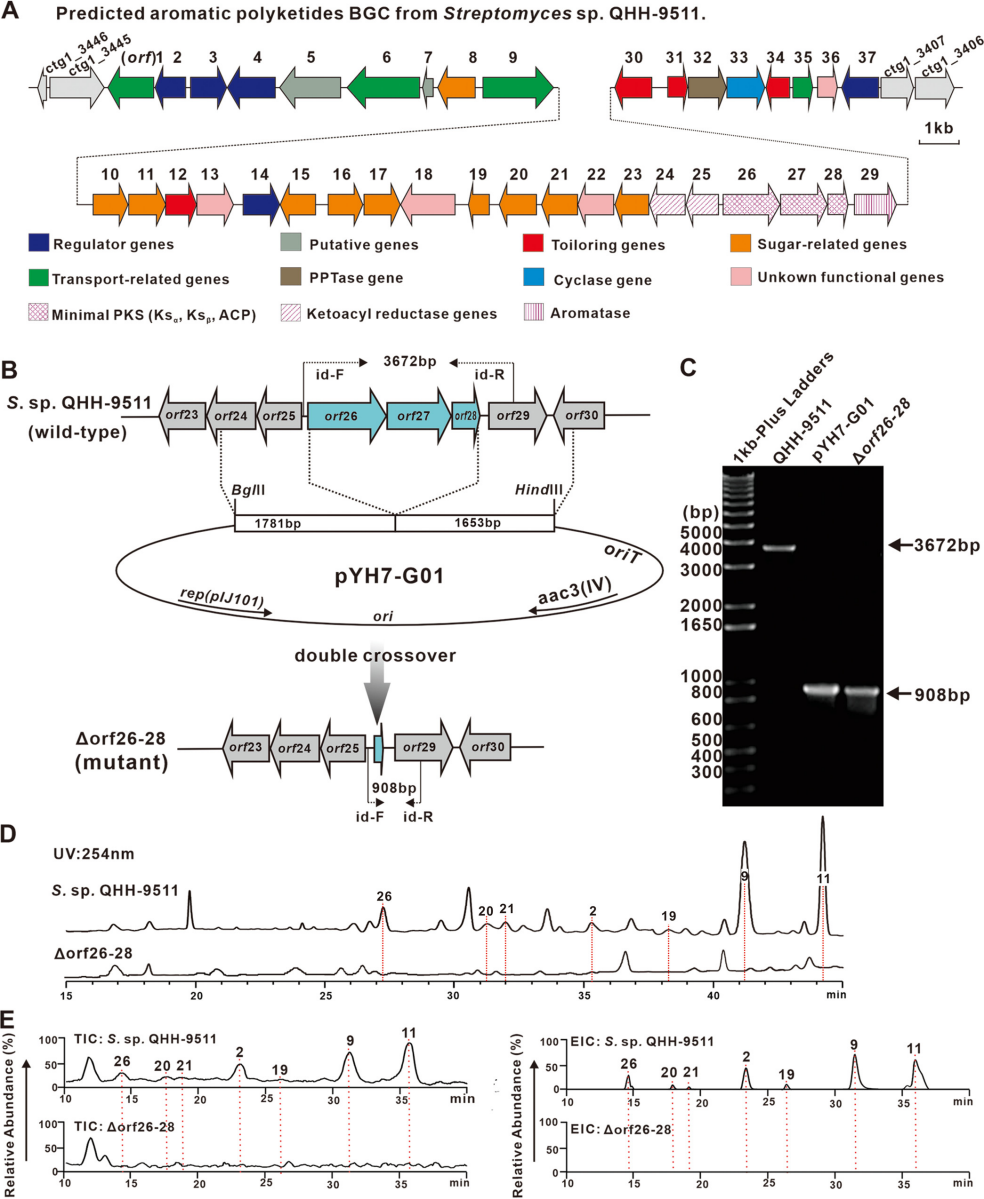
BGC identification of seven aromatic polyketides. (A) The arrows in the gene cluster framework schematic represent open readings frames (ORFs) involved in biosynthesis. ORFs for different functions are represented graphically with different colors and fills. For example, the purple oval represents a putative enzyme involved in the type II PKS system, and the blue oval represents the putative cyclase Orf33. Putative genes involved in the glycosylation pathway are shown in yellow. (B) Schematic representation of in-frame deletion of *orf*26-28 in *Streptomyces* sp. QHH-9511. (C) PCR confirmation of the mutation Δ*orf26*-*28*. (D) HPLC trace of aromatic polyketides from strain QHH-9511 and the mutant *Δorf26*-*28.* (E) LC-ESI-MS analysis of aromatic polyketides from strain QHH-9511 and the mutant *Δorf26*-*28*, and selective ion monitoring of *m/z* 233.08, 217.05, 305.10, 429.12, 231.07, 443.10, and 557.17 corresponding to compounds **2**, **9**, **11**, **19** to **21**, and **26**, respectively. TIC, total ion current. EIC, extracted ion current.

To identify the precise BGC(s) in strain QHH-9511 responsible for these seven compounds, cluster 20 (Fig. 6A, Table S6) was screened from three type II PKSs, because it contains multiple type II PKS-encoding genes and rhamnose-related modifier genes. Furthermore, gene cluster similarity analysis revealed that cluster 20 showed a high degree of similarity to the identified granaticin BGCs from Streptomyces violaceoruber Tü22 (38) and Streptomyces vietnamensis GIMV4.0001, as well as to gene clusters predicted from the genomes of other granaticin producers (Fig. S10). To verify the importance of the putative cluster, an in-frame deletion of *orf26-28* (putative keto-acyl synthase and acyl carrier protein) was constructed (Fig. 6B and C). HPLC analysis of the *Δorf26-28* mutant strain revealed abolishment of the seven compound traces relative to the wild-type strain (Fig. 6D), along with reduced anti-MRSA activity on a plate assay (Fig. S11), consistent with all seven compounds arising from the same BGC.

Further analysis using LC–electrospray ionization–high-resolution mass spectrometry (LC-ESI-HRMS) in negative ion mode gave total ion current (TIC) profiles showing abolition of product peaks in the *Δorf26-28* mutant compared to the wild type, and the extracted ion current (EIC) specifically indicated that target ions could not be detected in the mutant (Fig. 6E). These findings provided substantial evidence that the BGC is indeed responsible for the biosynthesis of these seven aromatic polyketides. This result broadens our knowledge of aromatic polyketide biosynthesis.

### Comparative genomic analysis of granaticin producers.

**FIG 7:**
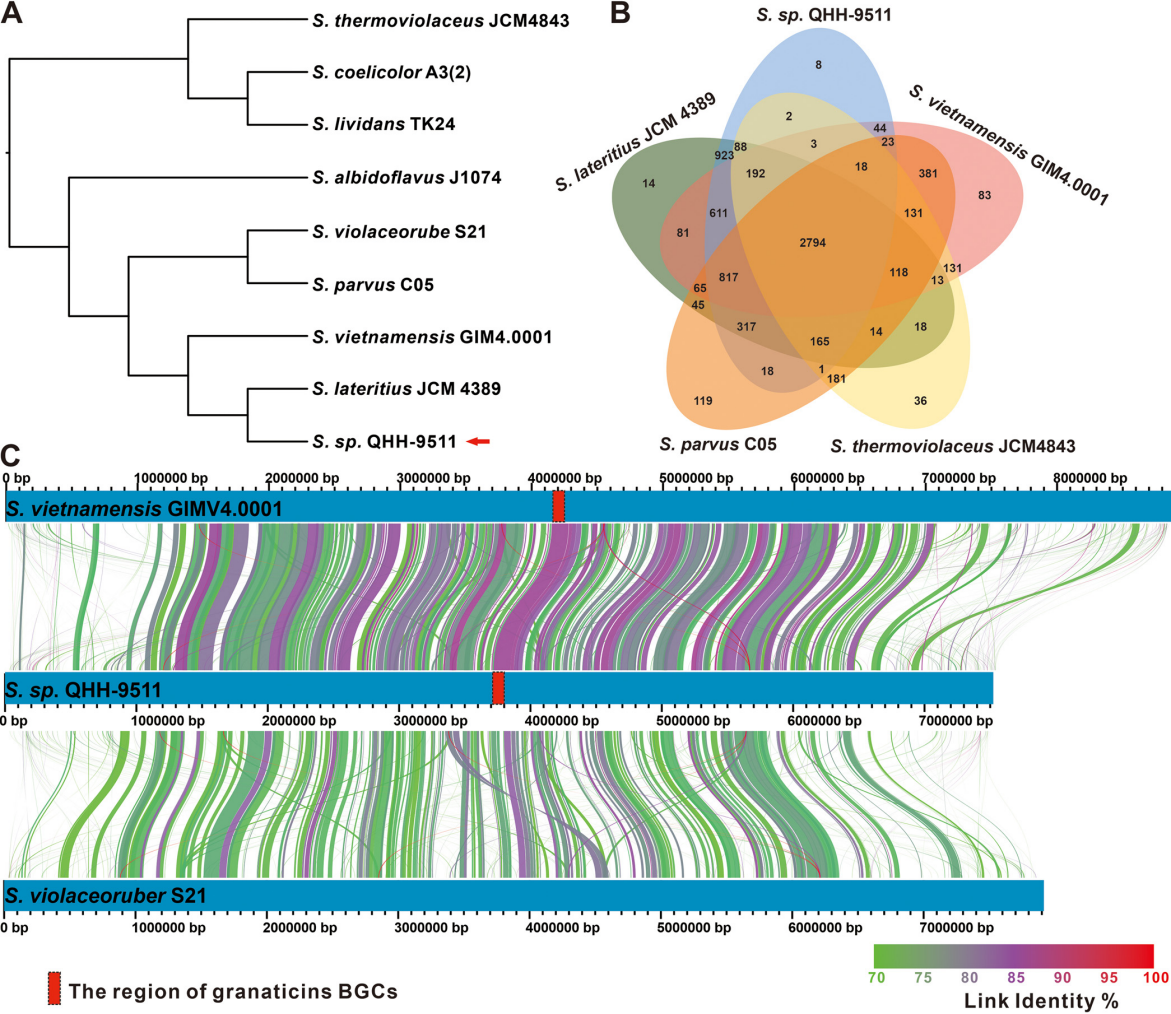
Comparative genomic analysis of granaticin producers. (A) Phylogenomic analysis of a granaticin producer and related strains based on 1,824 single-copy genes. (B) Venn diagram of orthologous groups of granaticin producer including strain QHH-9511. (C) Collinearity analysis of granaticins producers (GIMV4.0001 and QHH-9511) and strain S21.

Since their initial discovery in *S. olivaceus* (39), compounds **9** and **11** have been discovered in *S. violaceoruber* (36), thermophilic *S. thermoviolaceus* subsp. *pingens* var. WR-141(39) and strain NCIB 10076 (40), and *S. litmogenes* (41), as well as *S. vietnamensis* GIMV4.0001(42). The complete biosynthetic pathway of compounds **9** and **11** has been established in *S. violaceoruber* (38). The characterization of the source of the multistrain production of granaticins has stimulated our interest in the genomes of these producers. Phylogenomic analysis based on 1,824 single-copy genes showed that strains QHH-9511 and JCM 4389 were in a minimal branch, while JCM4843, a thermophilic granaticins producer, and QHH-9511 were the most evolutionarily distant (Fig. 7A). Further pan-genome analysis revealed that QHH-9511 shares 2,794 core genes with other granaticin producers, including genes related to granaticin biosynthesis. Furthermore, strain QHH-9511 contains eight unique genes, which is fewer than found in other producers (Fig. 7B). Collinearity analysis based on complete chromosomal genome sequences indicated that strains QHH-9511 and GIM4.0001 exhibited high similarity, far exceeding its similarity to *S. violaceoruber* S21. The BGC for granaticins (**9** and **11**) were in the region with the highest similarity between strains QHH-9511 and GIM4.0001, whereas the corresponding region in strain S21 did not contain the BGC for granaticins (Fig. 7C). The fact that granaticin BGCs are distributed among different sources of producers implies that granaticins may be a universal defense molecule produced by their producers for adaptation.

## DISCUSSION

Actinomycetes derived from special habitats have always been a rich source of a variety of active natural products, and microorganisms living in special environments often evolve their genomes in the process of adapting to their surroundings. The phylogenomic analysis of strain QHH-9511 demonstrated that its unique evolutionary state partially reflects this law. During their evolutionary process, microbes have developed various strategies for secondary metabolites biosynthesis to acclimatize to the surrounding environment. Generally, biosynthesis of a specific metabolite is controlled by a single BGC, which is almost distributed throughout the microbial community (43). For example, metabolites that are prevalent in actinomyces are also naturally present in strain QHH-9511, such as the five BGCs, which include cluster 3, which encodes alkylresorcinol. However, two-thirds (26) of the BGCs do not cluster to form GCF networks, reflecting the unpredictability of these gene clusters and highlighting the uniqueness of strain QHH-9511.

Theoretically, many actinomycetes have derived a variety of resistance mechanisms to adapt to extreme environments, such as low temperature, low oxygen, high salt, and high pressure, making their secondary metabolic pathways more specific and their product structures more novel. This is the main reason why researchers are keen to study the secondary metabolites of microorganisms. However, molecular network-based metabolite profiling of QHH-9511 did not reveal complex molecular clustering, nor did the isolation and structural identification of monomeric compounds lead to new molecules. This phenomenon is most likely due to the low yield of some potentially new molecules under current culture conditions, or because the metabolic potential of strain QHH-9511 cannot be activated by conventional cultivation conditions.

Although the aromatic polyketide compounds **2**, **9**, **11**, **19** to **21**, and **26** are all previously reported natural products, they are derived from different producers. Compound **2,** a proposed intermediate in the biosynthesis of granaticins (**9** and **11**), was initially isolated from *Streptomyces* sp. CPCC 200532 (35). Compounds **19** and **20** were first isolated from the recombinant S. lividans K4-114 containing actinorhodin polyketide synthase of S. coelicolor CH999 (33). The only published report on compund **21** comes from *Streptomyces* sp. Tü 6319 (34). Compound **26** was originally derived from *Micromonospora* sp. SA246 (44) and was later identified as the specific product of the actinomycin BGC in S. coelicolor A3(2) (37).

**FIG 8:**
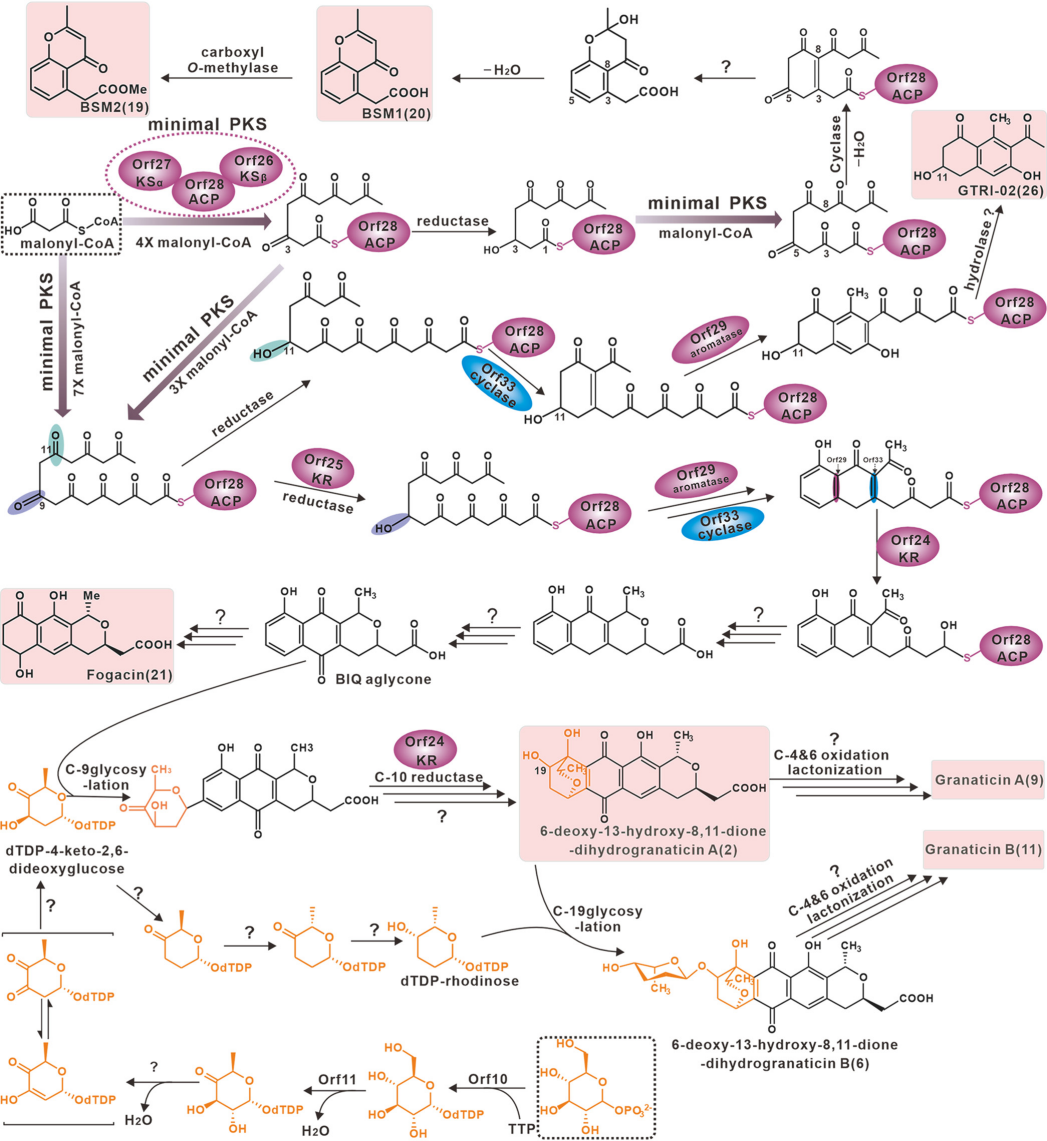
Proposed biosynthetic pathway of the aromatic polyketides. The dotted boxes indicate the initial substrate for the pathway, and the shaded boxes indicate the aromatic polyketides isolated from *Streptomyces* sp. QHH-9511. The purple ellipses represent the enzymes involved in the type II PKS system, and the blue ellipse represents the cyclase Orf33. The structure involved in the glycosylation pathway is shown in yellow.

Metabolic shunts are not rare in the biosynthesis of secondary metabolites and give rise to various products. Compound **26** and related polyketides as metabolic shunt products of the biosynthesis of **9** and **11** were revealed in *S. vietnamensis* GIMV4.0001 by time course metabolic profiling (45). The seven aromatic polyketides produced by strain QHH-9511 were all derived from the same BGC (cluster 20), and compounds **2** and **19** to **21** were also thought to be metabolic shunt products of the **9** and **11** biosynthetic pathways, according to genetic experiments aimed at disrupting the core function of the polyketide synthase. In terms of the biosynthetic pathway of BGC **20**, although the fluxes of malonyl-CoA are mostly directed to the octaketide chain of the end products **9** and **11**, hijacking of the polyketone chain by other enzymes from within or outside of the BGC during chain elongation may lead to short chain aromatic polyketides. Chain length factor determines the polyketide chain length (20), and enzymes capable of hijacking polyketone chain might display a weak competition with chain elongation factors in QHH-9511. For compounds **19** and **20**, the structures suggest that they are derived from the hexaketide intermediates that are reduced at the C-5 position, which indicates that the 5-ketoreduction reaction in the granaticin biosynthetic process occurs during polyketide chain elongation. We further speculate that the C-5 ketoreduction of the polyketone chain terminates its extension and directs it into the branch pathway (Fig. 8). For compound **26**, in contrast to traditional reduction at C-9, the C-11 reduction of the octaketide chain leads to the bypass pathway, and the subsequent two ring-forming aldol reactions set the backbone (Fig. 8). For compound **21**, its structural framework is more like that of actinorhodin than granaticins, whose subpathway may start as the BIQ aglycone (Fig. 8). It is interesting that an unusual metabolic shunt of a single BGC results in seven products with different chains. A possible explanation may be that imperfections in the minimal PKS machinery led to leakage of some shorter intermediates, which were then modified by enzymes or nonenzymatic reactions to generate the shunt products of granaticins.

The production of granaticins as the main product of strain QHH-9511 has been reported in several bacteria, including *S. olivaceus* from Angola soils (39), *Streptomyces thermophilus*, which produces grenadine more effectively when fermented at temperatures up to 45°C (40), and *S. vietnamensis* GIMV4.0001 from tropical rainforests (42). Strain QHH-9511 is the first granaticin producer from a lichen symbiosis at high altitudes. The BGC for granaticins has been identified in *S. violaceoruber* Tü 22 (38, 46) and *S. vietnamensis* GIMV4.0001 (42). The identification of the BGC for granaticins in QHH-9511 reveals the uniformity of origin of the seven aromatic polyketide compounds. An individual BGC choreographing the divergent biosynthesis of antibiotics with architecturally disparate skeletons might be more prevalent than previously thought. Considering the importance of secondary metabolites in nature, such divergent biosynthesis may offer an evolutionary advantage for the producer, allowing them to modify their biosynthetic strategies according to the changing survival pressures.

In summary, our initial goal was to look for anti-infective molecules from actinomycetes of specific habitats. Strain QHH-9511 appeared attractive to us due to its obvious antimicrobial metabolites, as well as its unique evolutionary status. BIG-SCAPE analysis also revealed that the vast majority of gene clusters on the genome were undescribed and nonclustered. These clues indicated that QHH-9511 is a novel actinomycete. Molecular network analysis of the metabolites of QHH-9511 revealed a large number of previously uncharacterized molecules, while separation and extraction of these molecules only identified a few common monomeric compounds. Subsequently, investigation into the structure-activity relationship of seven pigmental aromatic polyketides revealed the importance of specific functional groups. The convergent synthetic origin of these compounds was revealed by genetic experiments, indicating a diverse metabolic shunting of polyketide chains in strain QHH-9511. The current study is a comprehensive examination of the biosynthetic potential of lichen-derived *Streptomyces* on the Qinghai-Tibet Plateau at the genome and metabolite levels. The genome contains multiple unknown gene clusters and unstimulated metabolic potentials that need to be investigated further.

## MATERIALS AND METHODS

### Strains, culturing conditions, and DNA preparation.

Strain QHH-9511 was isolated from a yellow lichen on a stone surface on the west coast of Qing Hai Lake, and the specific isolation and purification method is described in a previous document (47). It was cultured for 3 days at 28°C on a Gauze’s agar plate. Then, a seed culture was maintained in liquid tryptic soy broth (TSB) medium at 30°C with shaking at 220 rpm for 3 days before being inoculated into Gauze's plate medium for solid fermentation for a week. High-quality genomic DNA was prepared with the salting out method.

### Genome sequencing, assembly, and annotation.

The whole-genome sequencing of QHH-9511 was performed using a combined strategy of Illumina Hiseq and PacBio RSII high-throughput sequencing technologies by Majorbio Corporation (Hangzhou, China), and we obtained 592-fold average genome coverage, with a paired-end library. SOAPdenovo 2.0425 (https://github.com/aquaskyline/SOAPdenovo2) assembled 33,853,158 readings from scratch. The PacBio platform sequencing data were corrected using BLASR26 (https://github.com/jcombs1/blasr) to map the Illumina sequencing reads and then assembled using the Celera assembler (http://wgs-assembler.sourceforge.net). Following the generation of a valid scaffold, the correction of sequencing reads was performed again using the Illumina data. The final finished assembly contained the entire genome sequence. Majorbio Cloud web platform was used to examine the basic genetic features. BLAST searches of nonredundant protein sequences from the NCBI, Swiss-Prot, COG, and KEGG databases were used to annotate the gene products' functions.

### Phylogenomic analyses and ANI calculations.

The phylogenomic comparison of the strain QHH-9511 genome with 624 annotated genomes of *Streptomyces* without duplication (https://www.ncbi.nlm.nih.gov/datasets/genomes/?taxon=1883&utm_source=gquery&utm_medium=referral) was completed by running OrthoFinder 2.5.4 (48) on a dual server equipped with E5-2699V4, which was visualized by using Figtree 1.4.4 (http://tree.bio.ed.ac.uk/software/figtree/). ANI values between the genomes of QHH-9511 and other species were calculated by FastANI (32).

The phylogenetic analysis of granaticin producers was performed with OrthoFinder 2.5.4 (48). The comparative genomic analysis based on orthologous genes was visualized by jvenn (http://jvenn.toulouse.inra.fr/app/example.html). Chromosomal genome and plasmid map visualization and genomic synteny analysis were achieved through MCScanX (49).

### Evaluation of secondary metabolite biosynthetic potentials.

The website antiSMASH 6.1 (https://antismash.secondarymetabolites.org/) was used to predict metabolite biosynthetic clusters, and further protein alignment and manual rechecking of the core genes within BGCs were performed. The predicted BGCs were subsequently detected as core or essential genes or antibiotic resistance genes by ARTS 2.0, and the predicted BGCs were further analyzed by BIG-SCAPE (11). Cutoffs was set to 0.75, the hits database was MIBiG14, and other parameters were default values. The final similarity network was visualized using Cytoscape 3.9.1.

### Secondary metabolite production, LC-DAD-MS detection, and GNPS analysis.

A seed culture containing QHH-9511 spores was kept at 30°C for 3 days in liquid TSB medium with 220 rpm shaking. Two different fermentation media were evaluated to optimize the chemical diversity of secondary metabolites produced by strain QHH-9511, including Gauze's plate medium and liquid medium (starch, 10 g; yeast extract, 4 g; peptone, 2 g; Fe_2_(SO_4_)_3_·4H_2_O, 40 mg; CaCO_3_, 1 g; KBr, 100 mg; H_2_O, 1,000 mL). Cultures were grown at 28°C for 7 days with shaking at 220 rpm. The violet-blue plate or liquid medium was collected separately, extracted three times with an equal volume of ethyl acetate (vol/vol), and the organic layer was concentrated *in vacuo* to give the crude extract.

Appropriate amounts of crude extracts from different fermentation methods were processed and detected by LC-DAD-MS. The electrospray mass spectra were recorded on an AB Sciex TripleTOF 6600 mass spectrometer in both positive-ion and negative-ion modes, and mass spectrometry data acquisition was done in IDA mode. The raw data were converted to .mzXML format before being uploaded to GNPS (gnps.ucsd.edu) for molecular network formation. To generate consensus spectra, the data were clustered with MS-cluster with a parent mass tolerance of 0.02 Da and an MS/MS fragment ion tolerance of 0.02 Da. The network file based on negative-ion mode MS data can be found and accessed at https://gnps.ucsd.edu/ProteoSAFe/status.jsp?task=c92dd9538224418781d24fb853be12ae. Cytoscape 3.9.1 was used to examine and visualize the network file.

### Isolation, identification, and activity evaluation of multiple compounds.

The violet-blue plate media were collected and extracted three times with an equal volume of ethyl acetate (vol/vol), and the organic layers were concentrated *in vacuo* to yield a brown ointment. The resulting extracts were then separated and purified with different types of chromatography, including silica gel, reversed-phase C_18_ column, and Sephadex LH-20. A detailed diagram of the separation process is shown in Fig. S12. Finally, totals of 29 compounds were separated on a semipreparative HPLC equipped with a Thermo Fisher Hypersil GOLD column (5 μm, 10 by 250 mm). The product from the wild type and mutant were detected with an Agilent HP1100 HPLC system equipped with a Phenomenex Luna C_18_ column (5 μm, 4.6 by 250 mm), with a linear gradient of protocol of 0 to 15 min, 5 to 30% B; 15 to 45 min, 30 to 75% B, at a flow rate of 0.6 mL/min (H_2_O for eluent A, and methanol [MeOH] for eluent B). UV absorption of HPLC-PDA was monitored at 254 nm. LC-ESI-HRMS analysis of the wild type and mutant were carried out on an LTQ XL Orbitrap (Thermo Fisher Scientific) coupled to an HPLC system, operated in negative-ion mode with electrospray ionization. HPLC separation was performed using the same Phenomenex column mentioned above. HPLC was equilibrated with H_2_O containing 20 mM ammonium formate (eluent A) and MeOH (eluent B), with the following program: 0 to 10 min, 5 to 25%B; 10 to 40 min, 25 to 80%B. The flow rate was 0.6 mL/min. Structural data for the compounds were obtained by using an AB Sciex TripleTOF 6600 mass spectrometer and/or Bruker Advance IIIHD 500-MHz NMR spectrometer.

MICs were determined in a dilution assay with the seven compounds using a series of pathogens, including methicillin-resistant Staphylococcus aureus ATCC 29231, Bacillus cereus IOP 01, Erwinia carotovora pv. *carotovora* NPCB 01, Erwinia carotovora subsp. *carotovora* (Jone) NPCB A3, Pseudomonas syringae NPCB 201, and Ralstonia solanacearum NPCB 002. The tests were done by taking 20 μL stock solution of each compound at 1 mg/mL in MeOH. Assays were conducted in 96-well microtiter plates in EBS medium (0.5% casein peptone, 0.5% glucose, 0.1% meat extract, 0.1% yeast extract, 50 mM HEPES [11.9 g/liter]; pH 7.0) for bacteria.

### HPLC analysis and LC-ESI-MS analysis.

The retention time characterization of seven aromatic compounds and the detection of mutant strains were performed by Agilent HP1100 HPLC equipped with a Phenomenex Luna C_18_ column (5 μm, 4.6 by 250 mm), with a linear gradient of protocol of 0 to 15 min, 5 to 30% B; 15 to 45 min, 30 to 75% B, at a flow rate of 0.6 mL/min (H_2_O for eluent A and MeOH for eluent B). UV absorption of HPLC-PDA was monitored at 254 nm.

LC-ESI-HRMS analyses of the mutant were carried out on an LTQ XL Orbitrap (Thermo Fisher Scientific) coupled to an HPLC system, operated in negative-ion mode with electrospray ionization. HPLC separation was performed using the same Phenomenex column mentioned above. HPLC was equilibrated with H_2_O containing 20 mM ammonium formate (eluent A) and MeOH (eluent B), with the following program: 0 to 10 min, 5 to 25% B; 10 to 40 min, 25 to 80% B. The flow rate was 0.6 mL/min.

### Genetic manipulation of the aromatic polyketide-producing strain.

First, the target gene region disruption plasmid pYH7-G01 was created in order to obtain target mutants to find BGCs for aromatic polyketides. The homologous arms were created by amplifying areas upstream and downstream of *orf26* (1.6 kb and 1.8 kb, respectively) with genomic DNA and the primer pairs Larm-F (5′-AGGTCGACCAAAGCGGCCATCGTGCCTCCCCACTCCTGCAtgctcaccgcgtaagggatc-3′)/Larm-R (5′-GGTGCGTCGGACGGCTTGGTCAGGCCGCCTCGGCCTGGGCGgcggtgagcaggtcccaga-3′) and Rarm-F (5′-gcccaggccgaggcggcctg-3′)/Rarm-R (5′-GTTATGCAGCGGAAAAGATCCGTCGACCTGCAGGCATGCAggagtgggggcggacgtgga-3′). The shuttle digest pYH7 was digested by BglII and HindIII, treated with alkaline phosphatase to obtain the linear fragment, and then purified by gel electrophoresis. According to the manufacturer’s protocol, the ligation between linear pYH7 and the amplification was carried out with the isothermal Gibson assembly method. Plasmid pYH7 was used for conjugational transfer after confirmation by sequencing. The recombinant plasmids were transformed into Escherichia coli ET12567/pUZ8002 cells, and pYH7-G01 was introduced into the wild type by conjugation on a serum-free medium containing 50 mg/mL apramycin and 25 mg/mL nalidixic acid. The double-crossover mutants were created by removing pYH7-G01 from the exconconjugants by numerous rounds of nonselective growth. PCR analysis was used to assess the integrity of the mutant *orf26-28* using the primer pair id-F(5′-GTCTCCCTTGGGTGGGGTGCG-3′) and idR (5′-GCCTCCTCGATACGGGCCCCGG-3′).

### Data availability.

The *Streptomyces* sp. QHH-9511 genome has been deposited in NCBI GenBank under accession numbers CP046905 and CP046906. All other data generated or analyzed during this study are included in this published article (and its supplemental material file).

## References

[1] DeLeo FR, Chambers HF. 2009. Reemergence of antibiotic-resistant *Staphylococcus aureus* in the genomics era. J Clin Invest 119:2464–2474. doi:10.1172/JCI38226. 19729844 PMC2735934

[2] Santos-Aberturas J, Vior NM. 2022. Beyond soil-dwelling Actinobacteria: fantastic antibiotics and where to find them. Antibiotics 11:195. doi:10.3390/antibiotics11020195. 35203798 PMC8868522

[3] Rosic NN. 2021. Recent advances in the discovery of novel marine natural products and mycosporine-like amino acid UV-absorbing compounds. Appl Microbiol Biotechnol 105:7053–7067. doi:10.1007/s00253-021-11467-9. 34480237 PMC8416575

[4] Quinn GA, Banat AM, Abdelhameed AM, Banat IM. 2020. *Streptomyces* from traditional medicine: sources of new innovations in antibiotic discovery. J Med Microbiol 69:1040–1048. doi:10.1099/jmm.0.001232. 32692643 PMC7642979

[5] Chevrette MG, Carlson CM, Ortega HE, Thomas C, Ananiev GE, Barns KJ, Book AJ, Cagnazzo J, Carlos C, Flanigan W, Grubbs KJ, Horn HA, Hoffmann FM, Klassen JL, Knack JJ, Lewin GR, McDonald BR, Muller L, Melo WGP, Pinto-Tomás AA, Schmitz A, Wendt-Pienkowski E, Wildman S, Zhao M, Zhang F, Bugni TS, Andes DR, Pupo MT, Currie CR. 2019. The antimicrobial potential of *Streptomyces* from insect microbiomes. Nat Commun 10:516. doi:10.1038/s41467-019-08438-0. 30705269 PMC6355912

[6] Bentley SD, Chater KF, Cerdeño-Tárraga AM, Challis GL, Thomson NR, James KD, Harris DE, Quail MA, Kieser H, Harper D, Bateman A, Brown S, Chandra G, Chen CW, Collins M, Cronin A, Fraser A, Goble A, Hidalgo J, Hornsby T, Howarth S, Huang CH, Kieser T, Larke L, Murphy L, Oliver K, O'Neil S, Rabbinowitsch E, Rajandream MA, Rutherford K, Rutter S, Seeger K, Saunders D, Sharp S, Squares R, Squares S, Taylor K, Warren T, Wietzorrek A, Woodward J, Barrell BG, Parkhill J, Hopwood DA. 2002. Complete genome sequence of the model actinomycete *Streptomyces coelicolor* A3(2). Nature 417:141–147. doi:10.1038/417141a. 12000953

[7] Ziemert N, Alanjary M, Weber T. 2016. The evolution of genome mining in microbes: a review. Nat Prod Rep 33:988–1005. doi:10.1039/c6np00025h. 27272205

[8] Machado H, Tuttle RN, Jensen PR. 2017. Omics-based natural product discovery and the lexicon of genome mining. Curr Opin Microbiol 39:136–142. doi:10.1016/j.mib.2017.10.025. 29175703 PMC5732065

[9] Thuan NH, Dhakal D, Pokhrel AR, Chu LL, Van Pham TT, Shrestha A, Sohng JK. 2018. Genome-guided exploration of metabolic features of *Streptomyces peucetius* ATCC 27952: past, current, and prospect. Appl Microbiol Biotechnol 102:4355–4370. doi:10.1007/s00253-018-8957-x. 29602983

[10] Hou S-Y, Zhang M-Y, Wang H-D, Zhang Y-X, Nojiri H. 2020. Biosynthesis gene cluster and oxazole ring formation enzyme for inthomycins in Streptomyces sp. strain SYP-A7193. Appl Environ Microbiol 86:e01388-20. doi:10.1128/AEM.01388-20. 32801183 PMC7531957

[11] Navarro-Muñoz JC, Selem-Mojica N, Mullowney MW, Kautsar SA, Tryon JH, Parkinson EI, De Los Santos ELC, Yeong M, Cruz-Morales P, Abubucker S, Roeters A, Lokhorst W, Fernandez-Guerra A, Cappelini LTD, Goering AW, Thomson RJ, Metcalf WW, Kelleher NL, Barona-Gomez F, Medema MH. 2020. A computational framework to explore large-scale biosynthetic diversity. Nat Chem Biol 16:60–68. doi:10.1038/s41589-019-0400-9. 31768033 PMC6917865

[12] Tettelin H, Masignani V, Cieslewicz MJ, Donati C, Medini D, Ward NL, Angiuoli SV, Crabtree J, Jones AL, Durkin AS, DeBoy RT, Davidsen TM, Mora M, Scarselli M, Margarit y Ros I, Peterson JD, Hauser CR, Sundaram JP, Nelson WC, Madupu R, Brinkac LM, Dodson RJ, Rosovitz MJ, Sullivan SA, Daugherty SC, Haft DH, Selengut J, Gwinn ML, Zhou L, Zafar N, Khouri H, Radune D, Dimitrov G, Watkins K, O'Connor KJB, Smith S, Utterback TR, White O, Rubens CE, Grandi G, Madoff LC, Kasper DL, Telford JL, Wessels MR, Rappuoli R, Fraser CM. 2005. Genome analysis of multiple pathogenic isolates of *Streptococcus agalactiae*: implications for the microbial pan-genome. Proc Natl Acad Sci USA 102:13950–13955. doi:10.1073/pnas.0506758102. 16172379 PMC1216834

[13] Read BA, Kegel J, Klute MJ, Kuo A, Lefebvre SC, Maumus F, Mayer C, Miller J, Monier A, Salamov A, Young J, Aguilar M, Claverie J-M, Frickenhaus S, Gonzalez K, Herman EK, Lin Y-C, Napier J, Ogata H, Sarno AF, Shmutz J, Schroeder D, de Vargas C, Verret F, von Dassow P, Valentin K, Van de Peer Y, Wheeler G, Allen AE, Bidle K, Borodovsky M, Bowler C, Brownlee C, Mark Cock J, Elias M, Gladyshev VN, Groth M, Guda C, Hadaegh A, Debora Iglesias-Rodriguez M, Jenkins J, Jones BM, Lawson T, Leese F, Lindquist E, Lobanov A, Lomsadze A, Malik S-B, Marsh ME, Mackinder L, Emiliania huxleyi Annotation Consortium, et al. 2013. Pan genome of the phytoplankton *Emiliania* underpins its global distribution. Nature 499:209–213. doi:10.1038/nature12221. 23760476

[14] Han J, Zhang J, Song Z, Liu M, Hu J, Hou C, Zhu G, Jiang L, Xia X, Quinn RJ, Feng Y, Zhang L, Hsiang T, Liu X. 2019. Genome- and MS-based mining of antibacterial chlorinated chromones and xanthones from the phytopathogenic fungus *Bipolaris sorokiniana* strain 11134. Appl Microbiol Biotechnol 103:5167–5181. doi:10.1007/s00253-019-09821-z. 31001746

[15] Chau R, Pearson LA, Cain J, Kalaitzis JA, Neilan BA, Atomi H. 2021. A *Pseudoalteromonas* clade with remarkable biosynthetic potential. Appl Environ Microbiol 87:e02604-20. doi:10.1128/AEM.02604-20. 33397702 PMC8105009

[16] Oberhofer M, Malfent F, Zehl M, Urban E, Wackerlig J, Reznicek G, Vignolle GA, Rückert C, Busche T, Wibberg D, Zotchev SB, Druzhinina IS. 2022. Biosynthetic potential of the endophytic fungus *Helotiales* sp. BL73 revealed via compound identification and genome mining. Appl Environ Microbiol 88:e02510-21. doi:10.1128/aem.02510-21. 35108081 PMC8939310

[17] Risdian C, Mozef T, Wink J. 2019. Biosynthesis of polyketides in *Streptomyces*. Microorganisms 7:124. doi:10.3390/microorganisms7050124. 31064143 PMC6560455

[18] Shen B. 2003. Polyketide biosynthesis beyond the type I, II and III polyketide synthase paradigms. Curr Opin Microbiol 7:285–295. doi:10.1016/S1367-5931(03)00020-6. 12714063

[19] Huffman MA, Fryszkowska A, Alvizo O, Borra-Garske M, Campos KR, Canada KA, Devine PN, Duan D, Forstater JH, Grosser ST, Halsey HM, Hughes GJ, Jo J, Joyce LA, Kolev JN, Liang J, Maloney KM, Mann BF, Marshall NM, McLaughlin M, Moore JC, Murphy GS, Nawrat CC, Nazor J, Novick S, Patel NR, Rodriguez-Granillo A, Robaire SA, Sherer EC, Truppo MD, Whittaker AM, Verma D, Xiao L, Xu Y, Yang H. 2019. Design of an *in vitro* biocatalytic cascade for the manufacture of islatravir. Science 366:1255–1259. doi:10.1126/science.aay8484. 31806816

[20] Tang Y, Tsai SC, Khosla C. 2003. Polyketide chain length control by chain length factor. J Am Chem Soc 125:12708–12709. doi:10.1021/ja0378759. 14558809

[21] Hertweck C, Luzhetskyy A, Rebets Y, Bechthold A. 2007. Type II polyketide synthases: gaining a deeper insight into enzymatic teamwork. Nat Prod Rep 24:162–190. doi:10.1039/b507395m. 17268612

[22] Shen Y, Yoon P, Yu TW, Floss HG, Hopwood D, Moore BS. 1999. Ectopic expression of the minimal whiE polyketide synthase generates a library of aromatic polyketides of diverse sizes and shapes. Proc Natl Acad Sci USA 96:3622–3627. doi:10.1073/pnas.96.7.3622. 10097087 PMC22344

[23] Terhi O, Kaisa P, Hanna L, Outi L, Kati H, Jarmo N, Pekka MN, Mikko MK. 2008. Characterization of the alnumycin gene cluster reveals unusual gene products for pyran ring formation and dioxan biosynthesis. Chem Biol 15:1046–1057. doi:10.1016/j.chembiol.2008.07.022. 18940666

[24] Wu C, Du C, Ichinose K, Choi YH, van Wezel GP. 2017. Discovery of C-glycosylpyranonaphthoquinones in *Streptomyces* sp. MBT76 by a combined NMR-based metabolomics and bioinformatics workflow. J Nat Prod 80:269–277. doi:10.1021/acs.jnatprod.6b00478. 28128554 PMC5373568

[25] Ichinose K, Ozawa M, Itou K, Kunieda K, Ebizuka Y. 2003. Cloning, sequencing and heterologous expression of the medermycin biosynthetic gene cluster of *Streptomyces* sp. AM-7161: towards comparative analysis of the benzoisochromanequinone gene clusters. Microbiology (Reading) 149:1633–1645. doi:10.1099/mic.0.26310-0. 12855716

[26] Fernandez-Moreno MA, Martinez E, Boto L, Hopwood DA, Malpartida F. 1992. Nucleotide sequence and deduced functions of a set of cotranscribed genes of *Streptomyces coelicolor* A3(2) including the polyketide synthase for the antibiotic actinorhodin. J Biol Chem 267:19278–19290. doi:10.1016/S0021-9258(18)41772-3. 1527048

[27] Sherman DH, Malpartida F, Bibb MJ, Kieser HM, Bibb MJ, Hopwood DA. 1989. Structure and deduced function of the granaticin-producing polyketide synthase gene cluster of *Streptomyces violaceoruber* Tü22. EMBO J 8:2717–2725. doi:10.1002/j.1460-2075.1989.tb08413.x. 2583128 PMC401279

[28] Naysmith BJ, Hume PA, Sperry J, Brimble MA. 2017. Pyranonaphthoquinones isolation, biology and synthesis: an update. Nat Prod Rep 34:25–61. doi:10.1039/c6np00080k. 27759131

[29] Usdin K. 2008. The biological effects of simple tandem repeats: lessons from the repeat expansion diseases. Genome Res 18:1011–1019. doi:10.1101/gr.070409.107. 18593815 PMC3960014

[30] Chater KF, Biro S, Lee KJ, Palmer T, Schrempf H. 2010. The complex extracellular biology of *Streptomyces*. FEMS Microbiol Rev 34:171–198. doi:10.1111/j.1574-6976.2009.00206.x. 20088961

[31] Ju K-S, Gao J, Doroghazi JR, Wang K-KA, Thibodeaux CJ, Li S, Metzger E, Fudala J, Su J, Zhang JK, Lee J, Cioni JP, Evans BS, Hirota R, Labeda DP, van der Donk WA, Metcalf WW. 2015. Discovery of phosphonic acid natural products by mining the genomes of 10,000 actinomycetes. Proc Natl Acad Sci USA 112:12175–12180. doi:10.1073/pnas.1500873112. 26324907 PMC4593130

[32] Jain C, Rodriguez-R LM, Phillippy AM, Konstantinidis KT, Aluru S. 2018. High throughput ANI analysis of 90K prokaryotic genomes reveals clear species boundaries. Nat Commun 9:5114. doi:10.1038/s41467-018-07641-9. 30504855 PMC6269478

[33] Kalaitzis JA, Moore BS. 2004. Heterologous biosynthesis of truncated hexaketides derived from the actinorhodin polyketide synthase. J Nat Prod 67:1419–1422. doi:10.1021/np0499564. 15332868

[34] Radzom M, Zeeck A, Antal N, Fiedler HP. 2006. Fogacin, a novel cyclic octaketide produced by *Streptomyces* strain Tu 6319. J Antibiot (Tokyo) 59:315–317. doi:10.1038/ja.2006.45. 16883783

[35] Jiang B, Li S, Zhao W, Li T, Zuo L, Nan Y, Wu L, Liu H, Yu L, Shan G, Zuo L. 2014. 6-Deoxy-13-hydroxy-8,11-dione-dihydrogranaticin B, an intermediate in granaticin biosynthesis, from *Streptomyces* sp. CPCC 200532. J Nat Prod 77:2130–2133. doi:10.1021/np500138k. 25153802

[36] Barcza S, Brufani M, Keller-Schierlein W, Zahner H. 1966. Metabolic products of microorganisms. 52. Granaticin B. Helv Chim Acta 49:1736–1740. doi:10.1002/hlca.19660490603. 5920453

[37] Wu C, Ichinose K, Choi Y, Wezel G. 2017. Aromatic polyketide GTRI-02 is a previously unidentified product of the act gene cluster in *Streptomyces coelicolor* A3(2). Chembiochem 18:1428–1434. doi:10.1002/cbic.201700107. 28463421

[38] Ichinose K, Bedford DJ, Tornus D, Bechthold A, Bibb MJ, Revill WP, Floss HG, Hopwood DA. 1998. The granaticin biosynthetic gene cluster of *Streptomyces violaceoruber* Tü22: sequence analysis and expression in a heterologous host. Chem Biol 5:647–659. doi:10.1016/S1074-5521(98)90292-7. 9831526

[39] Corbaz R, Ettlinger L, Gäumann E, Kalvoda J, Keller-Schierlein W, Kradolfer F, Manukian BK, Neipp L, Prelog V, Reusser P, Zähner H. 1957. Stoffwechselprodukte von Actinomyceten. 9. Mitteilung. Granaticin. HCA 40:1262–1269. doi:10.1002/hlca.19570400518.

[40] James PD, Edwards C. 1989. The effects of temperature on growth and production of the antibiotic granaticin by a thermotolerant streptomycete. J Gen Microbiol 135:1997–2003. doi:10.1099/00221287-135-7-1997. 2575655

[41] Chang C-J, Floss HG, Soong P, Chang C-T. 1975. Identity of the antitumor antibiotic litmomycin with granaticin A. J Antibiot (Tokyo) 28:156–156. doi:10.7164/antibiotics.28.156. 1112767

[42] Deng MR, Guo J, Zhu HH. 2011. *Streptomyces vietnamensis* GIMV4.0001: a granaticin-producing strain that can be readily genetically manipulated. J Antibiot (Tokyo) 64:345–347. doi:10.1038/ja.2011.3. 21304531

[43] Ostash B, Saghatelian A, Walker S. 2007. A streamlined metabolic pathway for the biosynthesis of moenomycin A. Chem Biol 14:257–267. doi:10.1016/j.chembiol.2007.01.008. 17379141 PMC1936435

[44] Yeo WH, Yun BS, Kim SS, Park EK, Kim YH, Yoo ID, Yu SH. 1998. GTRI-02, a new lipid peroxidation inhibitor from *Micromonospora* sp. SA246. J Antibiot (Tokyo) 51:952–953. doi:10.7164/antibiotics.51.952. 9917009

[45] Deng M-R, Li Y, He H-H, Zhou X, Zheng X-L, Wang Y-H, Zhu H. 2020. An aberrant metabolic flow toward early shunt products in the granaticin biosynthetic machinery of *Streptomyces vietnamensis* GIMV4.0001. J Antibiot (Tokyo) 73:260–264. doi:10.1038/s41429-019-0267-8. 31925390

[46] Bechthold A, Sohng JK, Smith TM, Chu X, Floss HG. 1995. Identification of *Streptomyces violaceoruber* Tü22 genes involved in the biosynthesis of granaticin. Mol Gen Genet 248:610–620. doi:10.1007/BF02423457. 7476861

[47] Hei Y, Zhang H, Tan N, Zhou Y, Wei X, Hu C, Liu Y, Wang L, Qi J, Gao J-M. 2021. Antimicrobial activity and biosynthetic potential of cultivable *actinomycetes* associated with Lichen symbiosis from Qinghai-Tibet Plateau. Microbiol Res 244:126652. doi:10.1016/j.micres.2020.126652. 33310352

[48] Emms DM, Kelly S. 2019. OrthoFinder: phylogenetic orthology inference for comparative genomics. Genome Biol 20:238. doi:10.1186/s13059-019-1832-y. 31727128 PMC6857279

[49] Wang Y, Tang H, Debarry JD, Tan X, Li J, Wang X, Lee TH, Jin H, Marler B, Guo H, Kissinger JC, Paterson AH. 2012. MCScanX: a toolkit for detection and evolutionary analysis of gene synteny and collinearity. Nucleic Acids Res 40:e49. doi:10.1093/nar/gkr1293. 22217600 PMC3326336

