# Supplementary material for: Genomic and Metabolite Profiling Reveal a Novel Streptomyces Strain, QHH-9511, from the Qinghai-Tibet Plateau
Source: Microbiol Spectr. 2023 Jan 9;11(1):e02764-22. doi: 10.1128/spectrum.02764-22 (PMC9927492; doi:10.1128/spectrum.02764-22)
Supplement: Supplemental file 1 — Supplemental text, Fig. S1 to S12, and Tables S1 to S6. Download spectrum.02764-22-s0001.pdf, PDF file, 4.8 MB [file spectrum.02764-22-s0001.pdf]

Supplementary Material

Genomic and metabolite profiling reveals a novel *Streptomyces* strain, QHH-9511, from a lichen symbiont on the Qinghai-Tibet Plateau

Xi-long Feng[a#], Rui-qi Zhang[a#], Da-cheng Wang[a], Wei-ge Dong[a], Zhen-xin Wang [a], Yi-jie Zhai[a], Wen-bo Han[a], Xia Yin[a], Junmian Tian[a], Jing Wei[bc], Jin-ming Gao[a], Jianzhao Qi[a]*

[a] Shaanxi Key Laboratory of Natural Products & Chemical Biology, College of Chemistry & Pharmacy, Northwest A&F University, 3 Taicheng Road, Yangling 712100, Shaanxi, China

[b] College of Biology Pharmacy & Food Engineering, Shangluo University, Shangluo 726000, Shaanxi, China

[c] Qinba Mountains of Bio-Resource Collaborative Innovation Center of Southern Shaanxi Province, Hanzhong 723000, Shaanxi, China

[#] These authors contributed equally to this paper.

*Corresponding author (Tel: +86-29-87092381 E-mail: qjz@nwafu.edu.cn)

CONTENT

1. General methods 1

1.1 Bacterial Strains and Culture Conditions 1

1.2 Bioassay and MIC Analysis 1

2. Supplementary Results 2

Figure S1. Characteristics of the strain QHH-9511 and its metabolite. 2

Figure S2. Gene length & frequency of genome from the strain QHH-9511 3

Figure S3. The GO annotation (A) & COG distribution (B) of all identified genes in the strain QHH-9511...4

Figure S4. The KEGG annotation of all identified genes in the strain QHH-9511. 5

Figure S5. GCF networks analysis of highly similar antiSMASH-based predicted BGCs.......6

Figure S6. HPLC-DAD fingerprint analysis of secondary metabolites produced by the strain QHH-9511 in solid fermentation (A) and liquid fermentation (B)................................7

Figure S7. Molecular network analysis for the metabolites of strain QHH- 9511..................8

Figure S8. The LC-ESI-HRMS and LC-ESI-HRMS/MS spectrums of isolates from the strain QHH- 9511 (TOF, negative mode)...10

Figure S9. The NMR spectrums of isolates from the strain QHH-9511. 14

Figure S10. Similarity comparison of identified, and unidentified granaticins BGCs, as well as related BGCs...15

Figure S11. Antimicrobial assay of QHH-9511 and the mutate Δ*orf26-28*. 16

Figure S12. Schematic diagram of the compound separation process. 17

Table S1| General features of the chromosome of the strain QHH-9511. 18

Table S2| Genomic islands (GIs) distributed on the genome of the strain QHH-9511...........19

Table S3| BGC Proximity (A) and Resistance models (B) in ARTS2.0 analysis of the strain QHH-9511..20

Table S4| The NMR data of several aromatic polyketides. 22

Table S5| The minimum inhibitory concentration test for seven compounds using human pathogen and phytopathogen...24

Table S6| Deduced functions of the open reading frames of the putative granaticins cluster in the strain QHH-9511. 25

3. References 26

1. General methods

1.1 Bacterial Strains and Culture Conditions

Streptomycete sp. QHH-9511 and mutant strain Δorf26-28 were grown in TSB medium for collection thallus or of seed cultivation, and on SFM(1) medium for spore germination and conjugation. *E. coli* DH10B and ET12567/pUZ8002 (2) strains were grown in Luria-Bertani liquid or ager medium at 37°C with appropriate antibiotic screening (chloramphenicol 25 μg/mL, apramycin 50 μg/mL, kanamycin 50 μg/mL).

1.2 Bioassay and MIC Analysis

The human and agricultural pathogens used in the tests were all classical indicator bacteria kept at the Institute of Pesticide Research, School of Plant Protection, Northwest Agriculture and Forestry University, and preserved in our laboratory by their gift. Methicillin-resistant and sensitive *Staphyloccocus aureus* were purchased from ATCC and stored in our laboratory, Shaanxi Key Laboratory of Natural Products & Chemical Biology.

For the determination of the minimal inhibitory concentrations (MIC), two-fold dilutions ranging from 0.125 to 256 mg/mL were used for pathogenic bacteria and fungal strains. For bacterial strains, 20 mL of each compound solution was added into 180 mL of LB medium in 96-well plates, and 1-5 $\times 10^5$ CFU were added. Growth was recorded after 20 h incubation at 30°C. The absorbance was measured by a microplate reader under OD_{600}. For filamentous fungi, 2 mL certain concentrations of compound solutions were added to 18 mL PDA medium plates. The plates were then kept at 4°C for 24 h to allow the diffusion of antibiotics. The fungi were then inoculated on the surface of the agar and incubated at 28°C for 48 h.

2. Supplementary Results

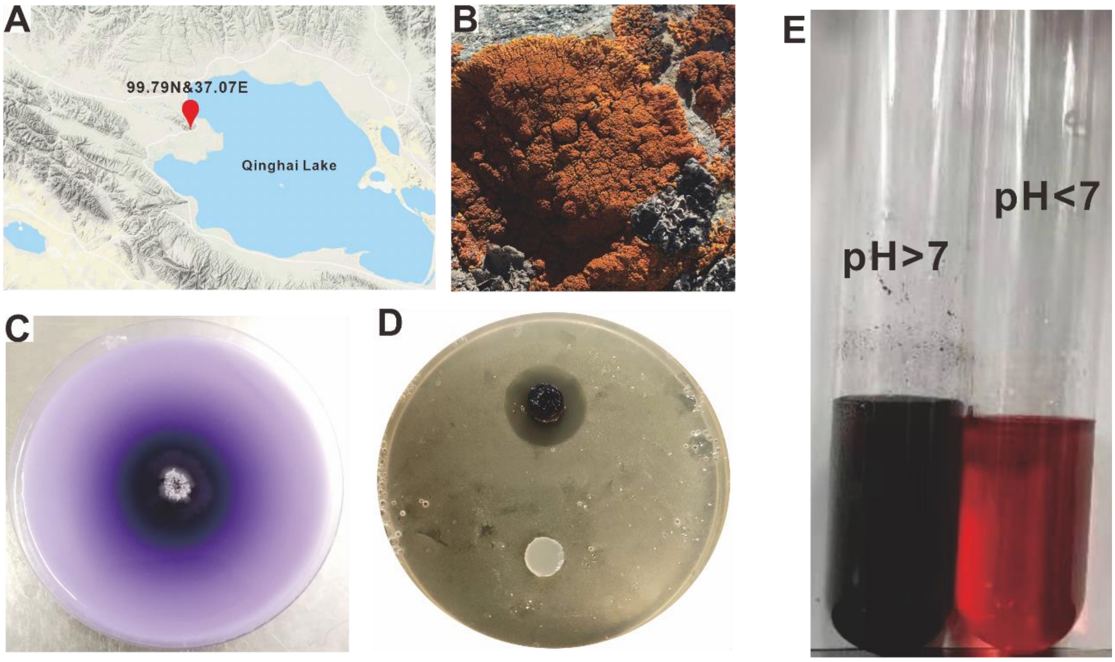

Figure S1. Characteristics of the strain QHH-9511 and its metabolite.
A: Sample collected site; B: A yellow lichen on the surface of a stone; C: Gauze's plate on which filaments grow for three days; D: Biological activity assay with MRSA(ATCC43300) as the indicator; E: The extracts of raw metabolite dissolved in water show different colors, blue-purple(pH>7) and blood-red(pH<7), respectively.

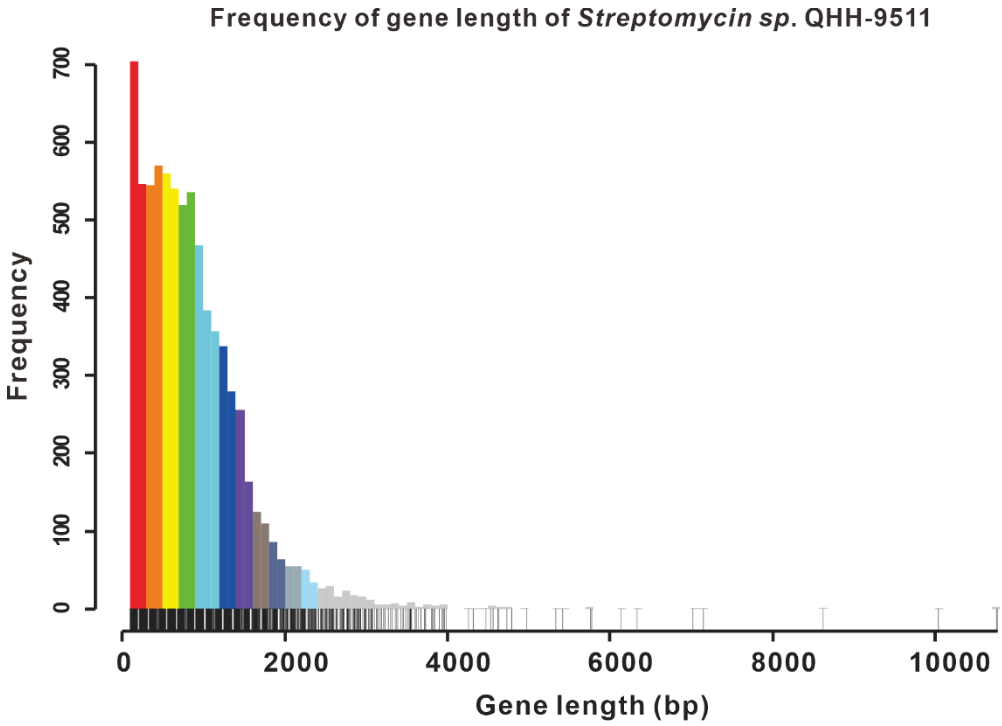

Figure S2. Gene length & frequency of genome from the strain QHH-9511.

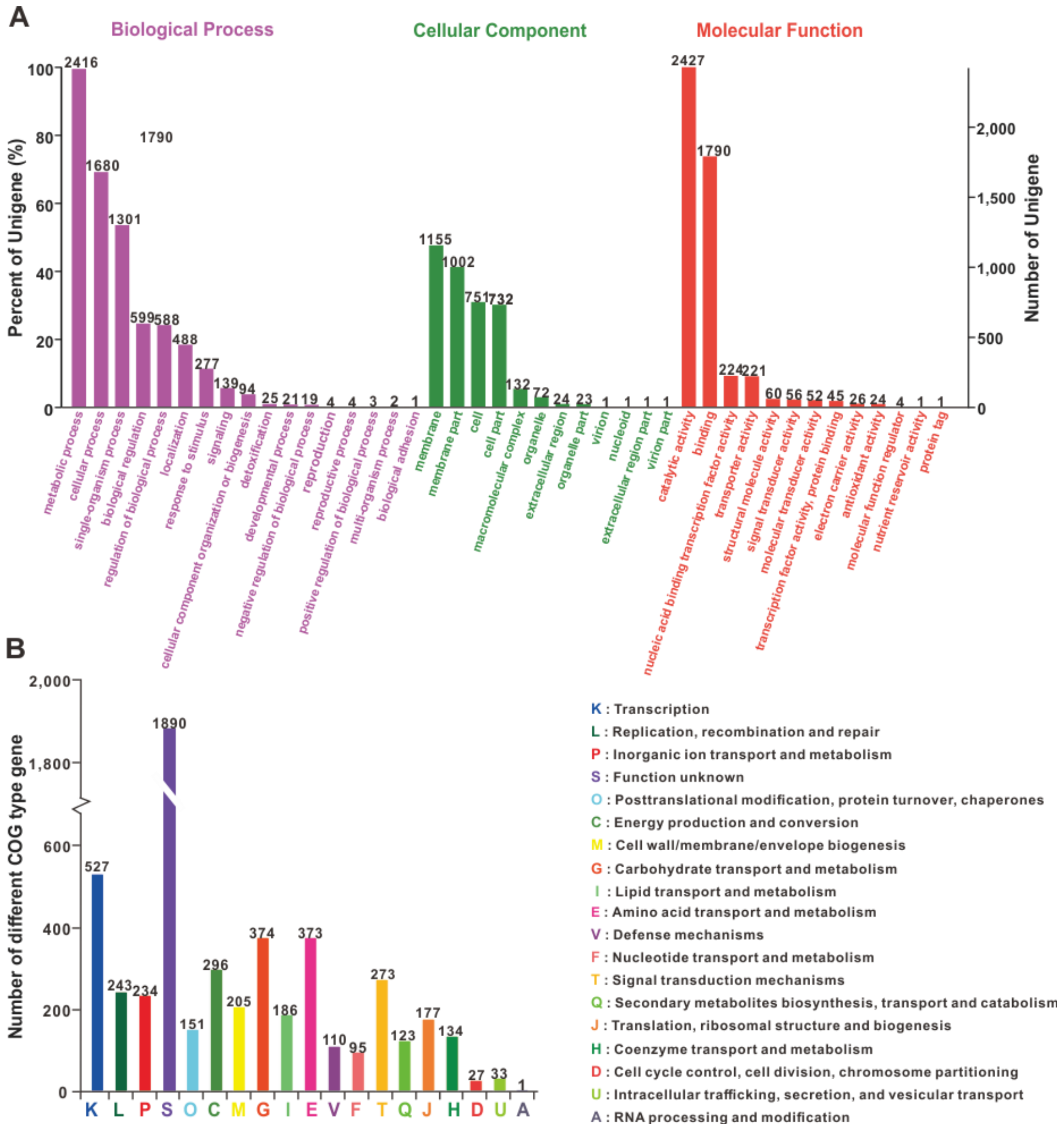

Figure S3. The GO annotation (A) & COG distribution (B) of all identified genes in the strain QHH-9511.

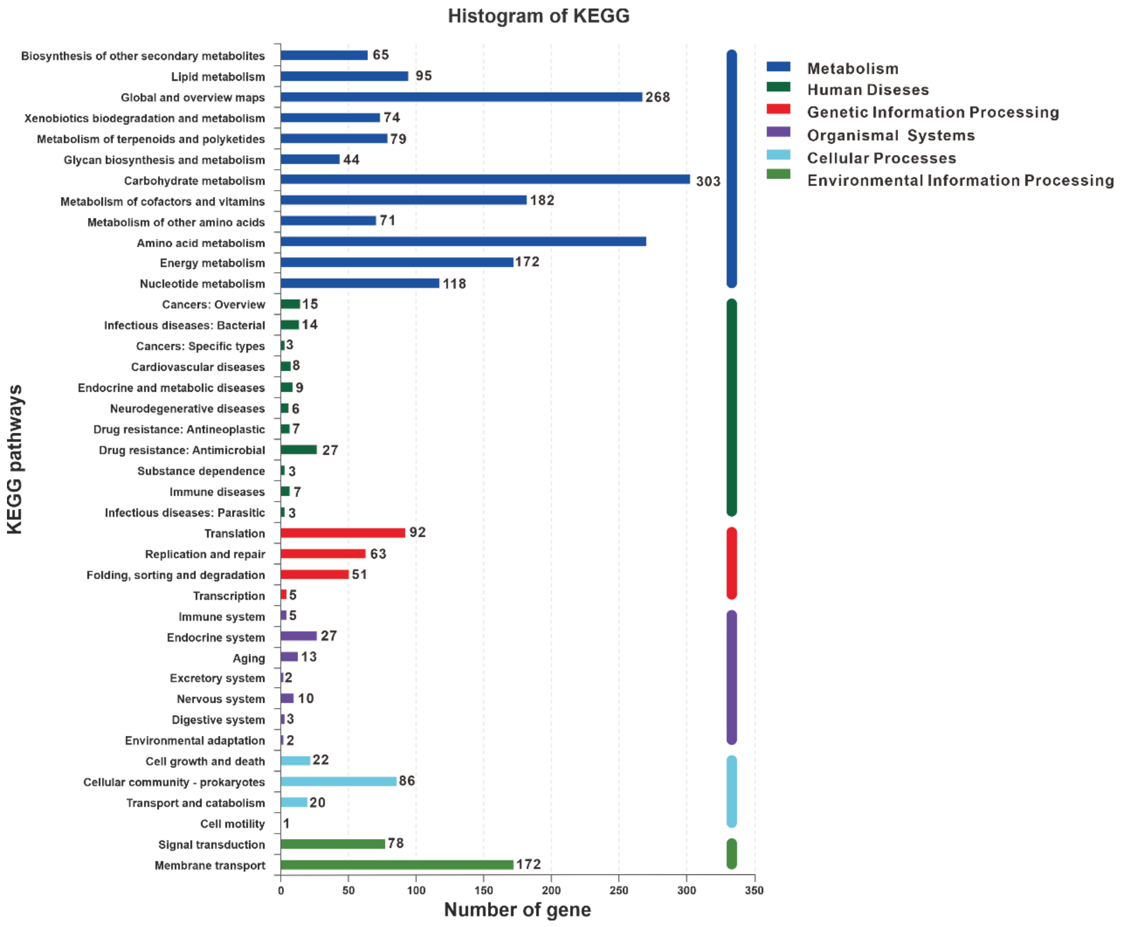

Figure S4. The KEGG annotation of all identified genes in the strain QHH-9511.

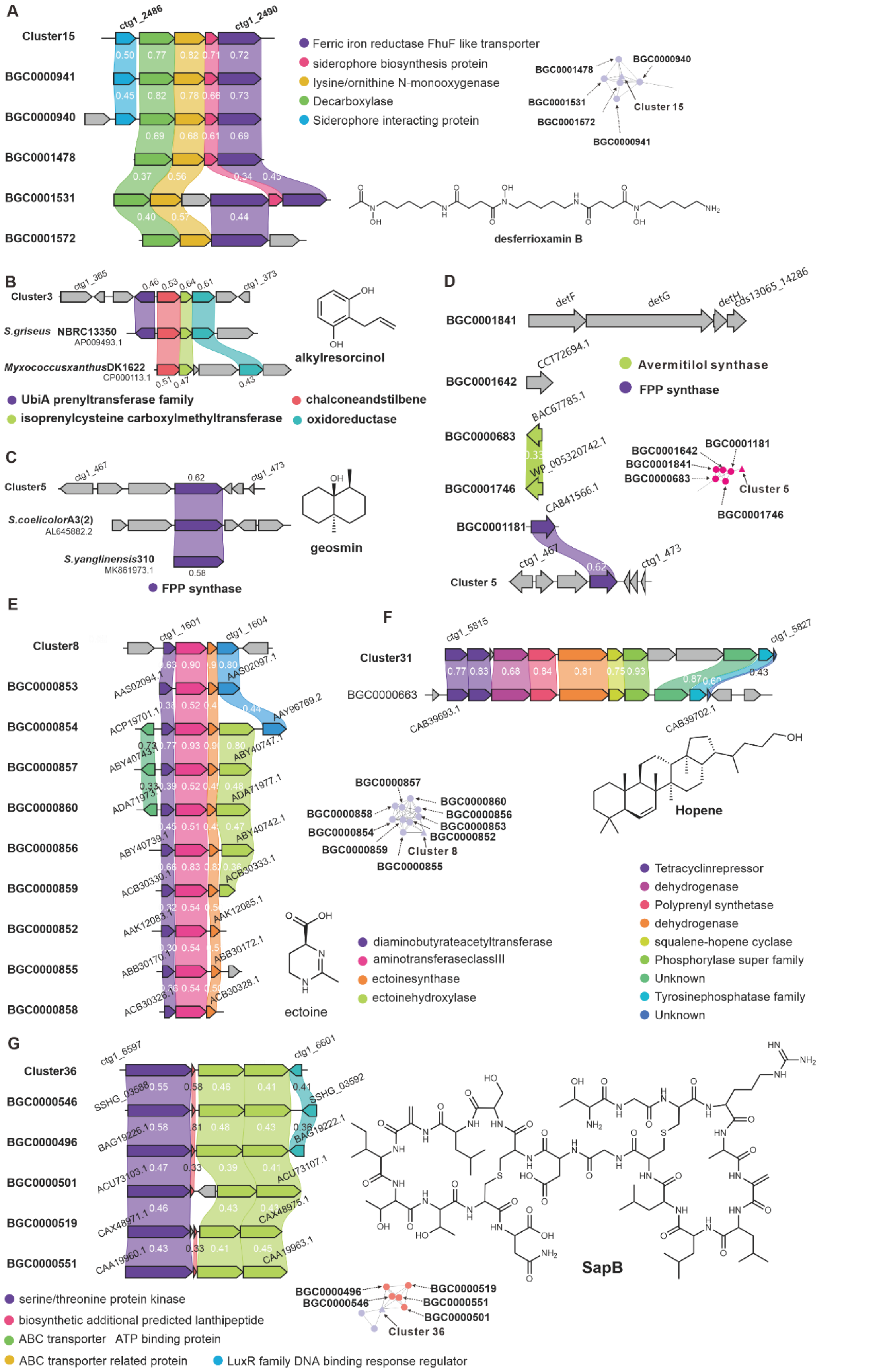

Figure S5. GCF networks analysis of highly similar antiSMASH-based predicted BGCs.

Similarity analysis of gene clusters was performed by clinker.

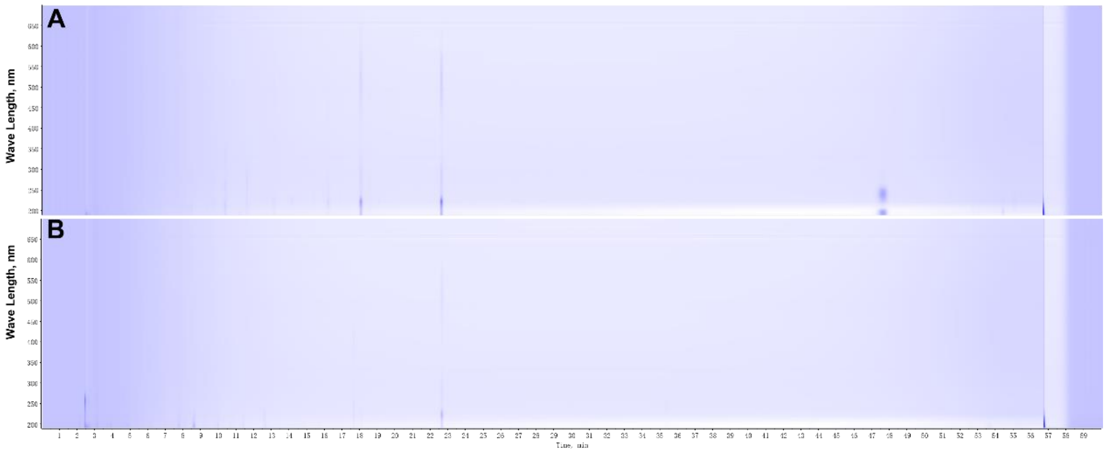

Figure S6. HPLC-DAD fingerprint analysis of secondary metabolites produced by the strain QHH-9511 in solid fermentation (A) and liquid fermentation (B).

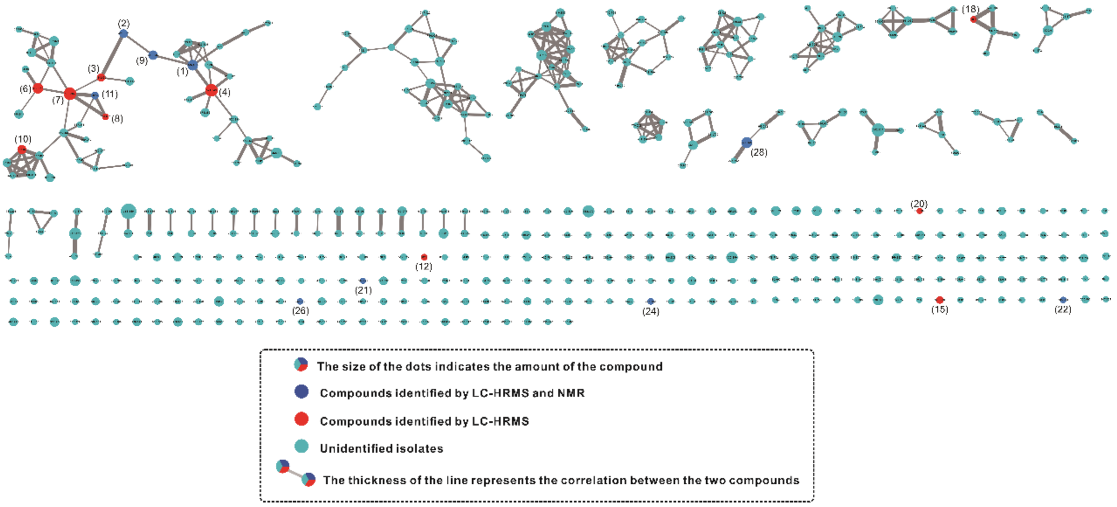

Figure S7. Molecular network analysis for the metabolites of strain QHH-9511.

Cosine similarity score cut-off: 0.70, generated using Cytoscape v3.9.1, each node is labeled by mass-to-charge ratio and the size of each node indicates its intensity.

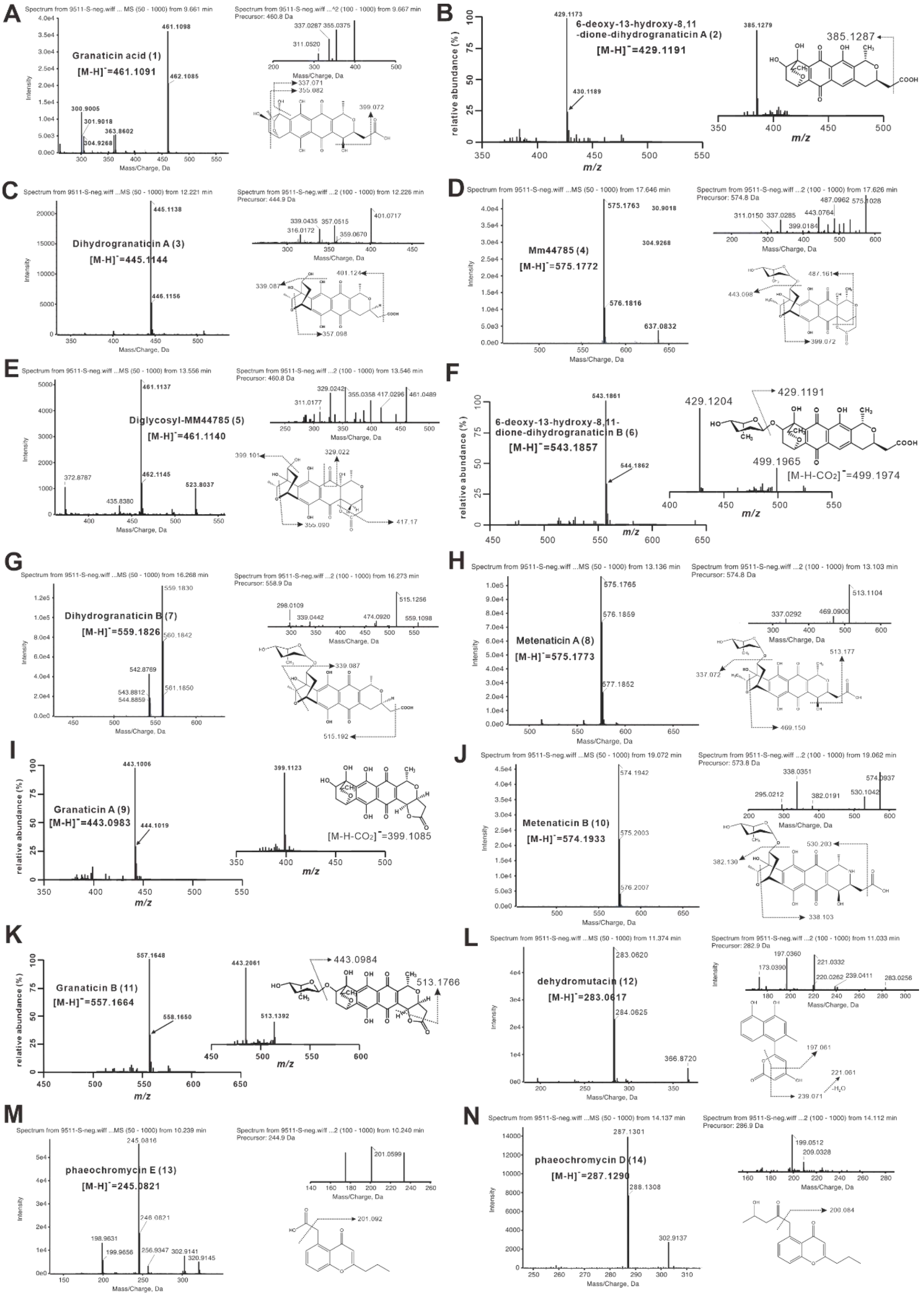
A
Granaticin acid (1)
[M-H]⁻=461.1091
B
6-deoxy-13-hydroxy-8,11 -dione-dihydrogranaticin A (2)
[M-H]⁻=429.1191
C
Dihydrogranaticin A (3)
[M-H]⁻=445.1144
D
Mm44785 (4)
[M-H]⁻=575.1772
E
Diglycosyl-MM44785 (5)
[M-H]⁻=461.1140
F
6-deoxy-13-hydroxy-8,11-dione-dihydrogranaticin B (6)
[M-H]⁻=543.1857
[M-H-CO2]⁻=499.1974
G
Dihydrogranaticin B (7)
[M-H]⁻=559.1826
H
Metenaticin A (8)
[M-H]⁻=575.1773
I
Granaticin A (9)
[M-H]⁻=443.0983
[M-H-CO2]⁻=399.1085
J
Metenaticin B (10)
[M-H]⁻=574.1933
K
Granaticin B (11)
[M-H]⁻=557.1664
L
dehydromutacin (12)
[M-H]⁻=283.0617
M
phaeochromycin E (13)
[M-H]⁻=245.0821
N
phaeochromycin D (14)
[M-H]⁻=287.1290
relative abundance (%)
m/z
Intensity
Mass/Charge, Da

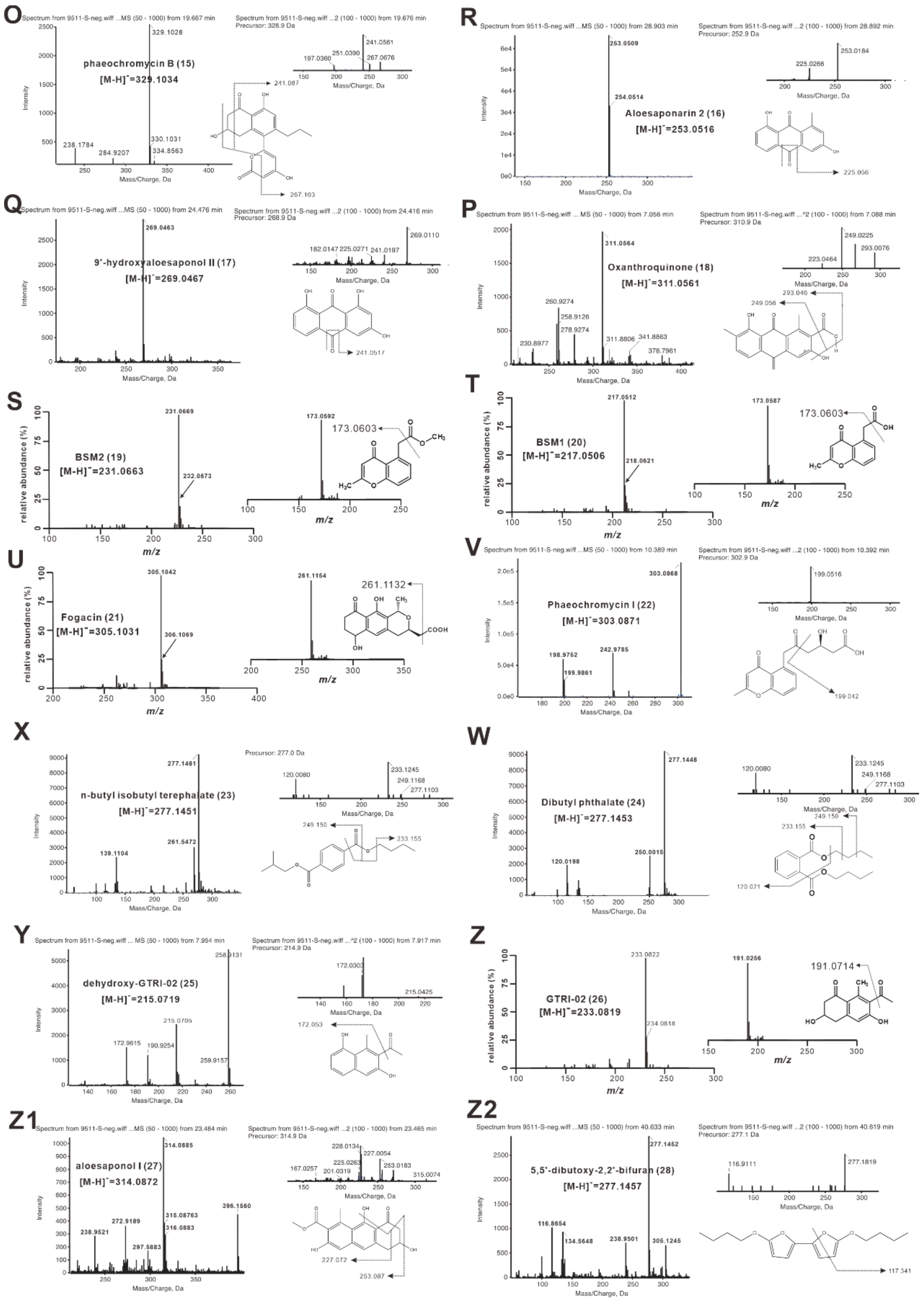

Figure S8. The LC-ESI-HRMS and LC-ESI-HRMS/MS spectrums of isolates from the strain QHH-9511 (TOF, negative mode).

Numbers A-Z2 correspond to compounds 1-28.

I

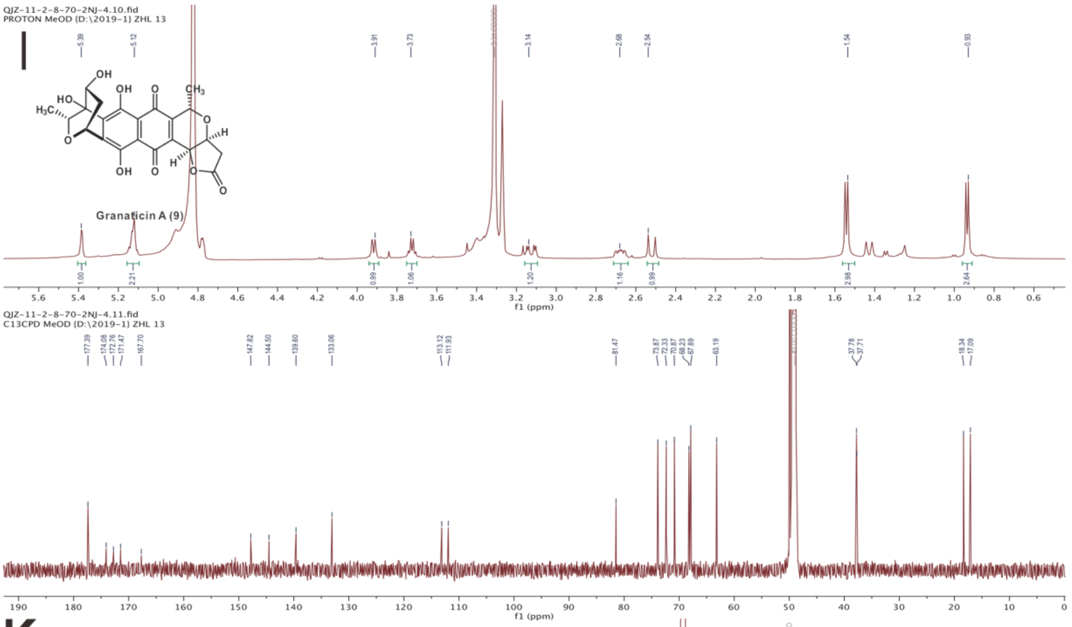
QJZ-11-2-8-70-2NJ-4.10.fid
PROTON MeOD (D:\2019-1) ZHL 13
Granaticin A (9)
f1 (ppm)
QJZ-11-2-8-70-2NJ-4.11.fid
C13CPD MeOD (D:\2019-1) ZHL 13
f1 (ppm)

K

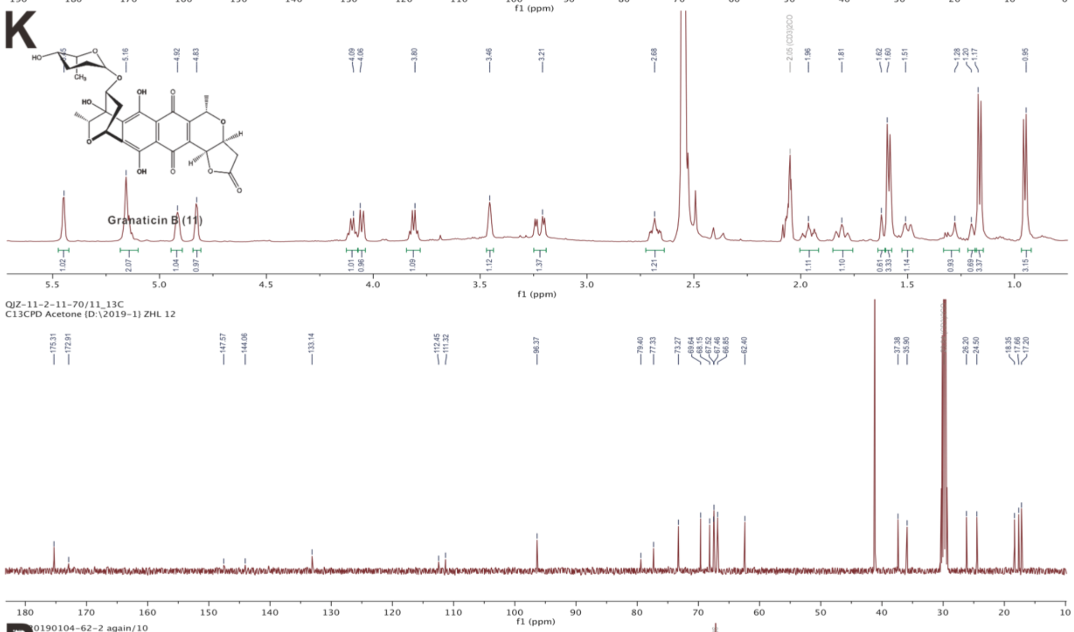
Granaticin B (11)
f1 (ppm)
QJZ-11-2-11-70/11_13C
C13CPD Acetone (D:\2019-1) ZHL 12
f1 (ppm)

P

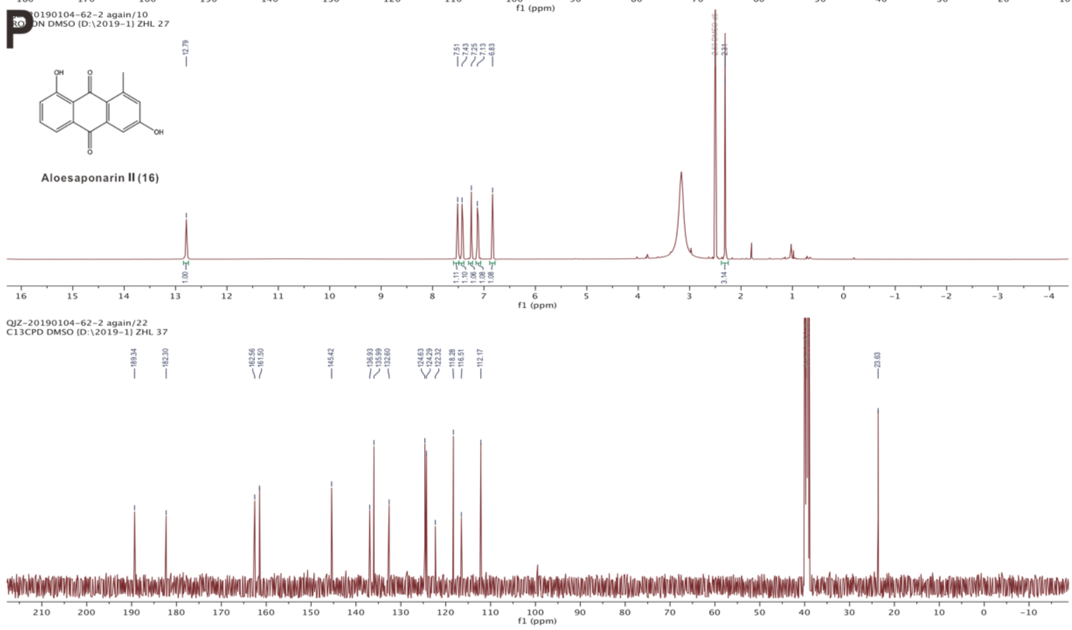
20190104-62-2 again/10
DN DMSO (D:\2019-1) ZHL 27
Aloesaponarin II (16)
f1 (ppm)
QJZ-20190104-62-2 again/22
C13CPD DMSO (D:\2019-1) ZHL 37
f1 (ppm)

S

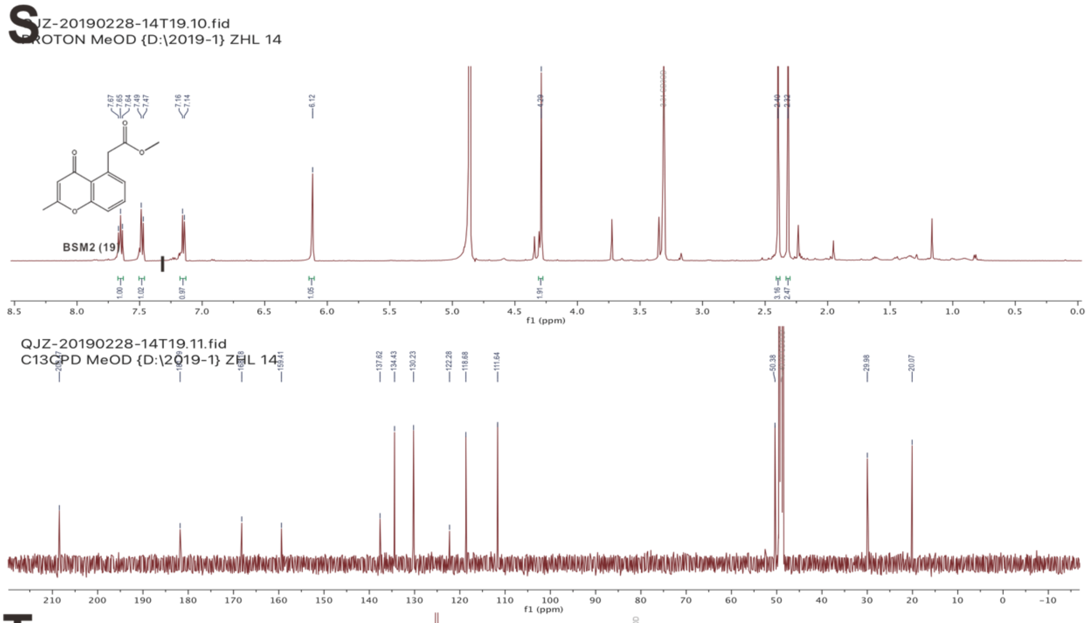
JZ-20190228-14T19.10.fid
ROTON MeOD {D:\2019-1} ZHL 14
BSM2 (19)
f1 (ppm)
QJZ-20190228-14T19.11.fid
C13CPD MeOD {D:\2019-1} ZHL 14
f1 (ppm)

T

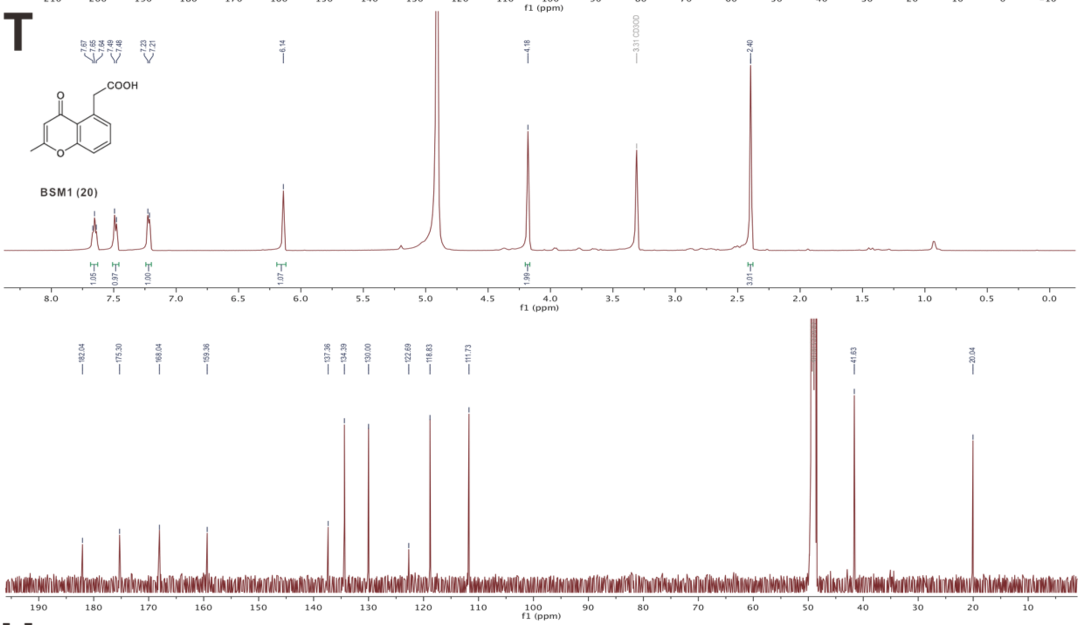
COOH
BSM1 (20)
f1 (ppm)
f1 (ppm)

U

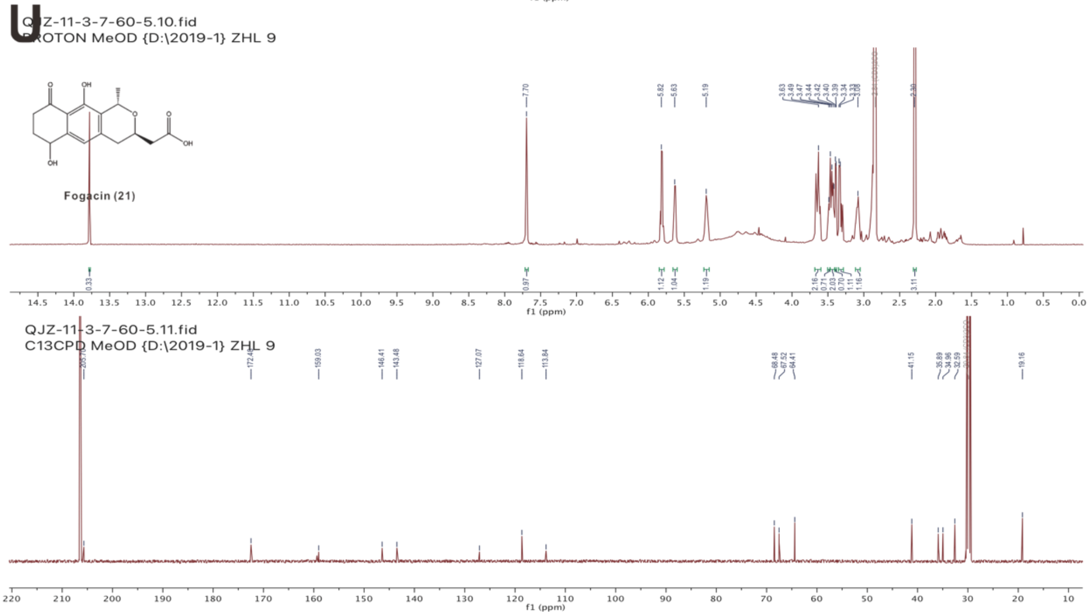
Z-11-3-7-60-5.10.fid
ROTON MeOD {D:\2019-1} ZHL 9
Fogacin (21)
f1 (ppm)
QJZ-11-3-7-60-5.11.fid
C13CPD MeOD {D:\2019-1} ZHL 9
f1 (ppm)

V

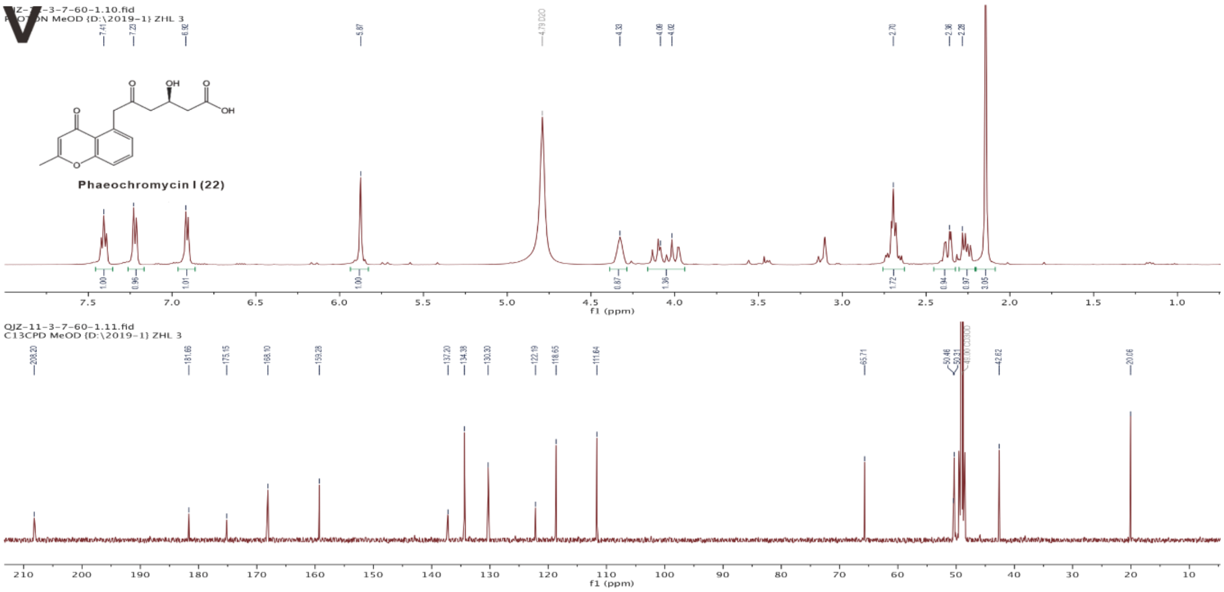
Phaeochromycin I (22)
QJZ-11-3-7-60-1.11.fid
C13CPD MeOD {D:\2019-1} ZHL 3
f1 (ppm)

W

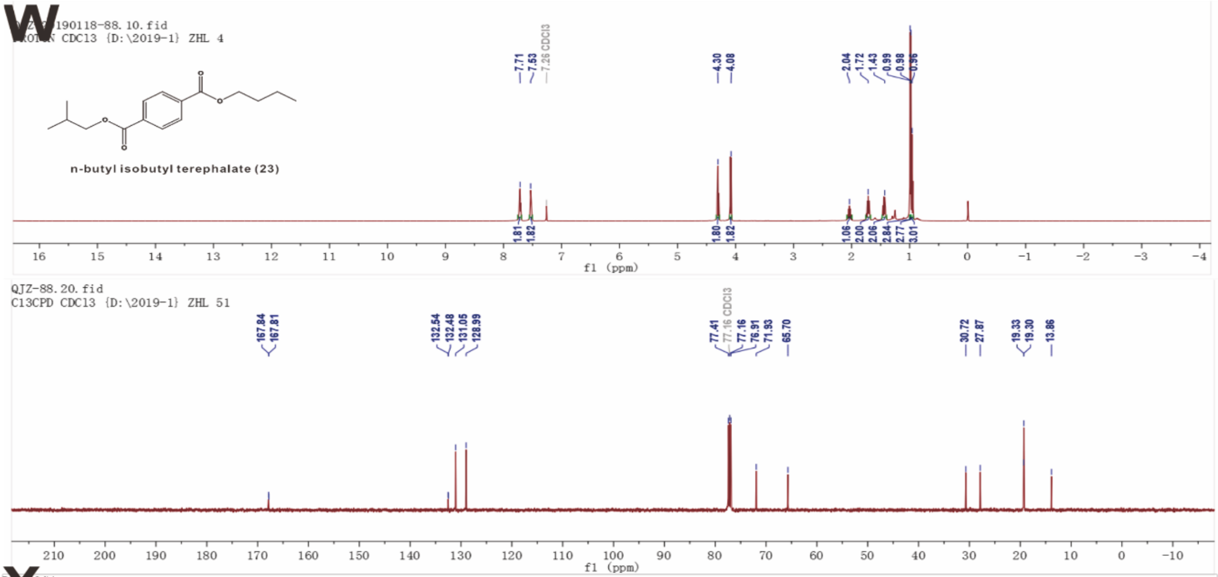
n-butyl isobutyl terephalate (23)
QJZ-88.20.fid
C13CPD CDC13 {D:\2019-1} ZHL 51
f1 (ppm)

X

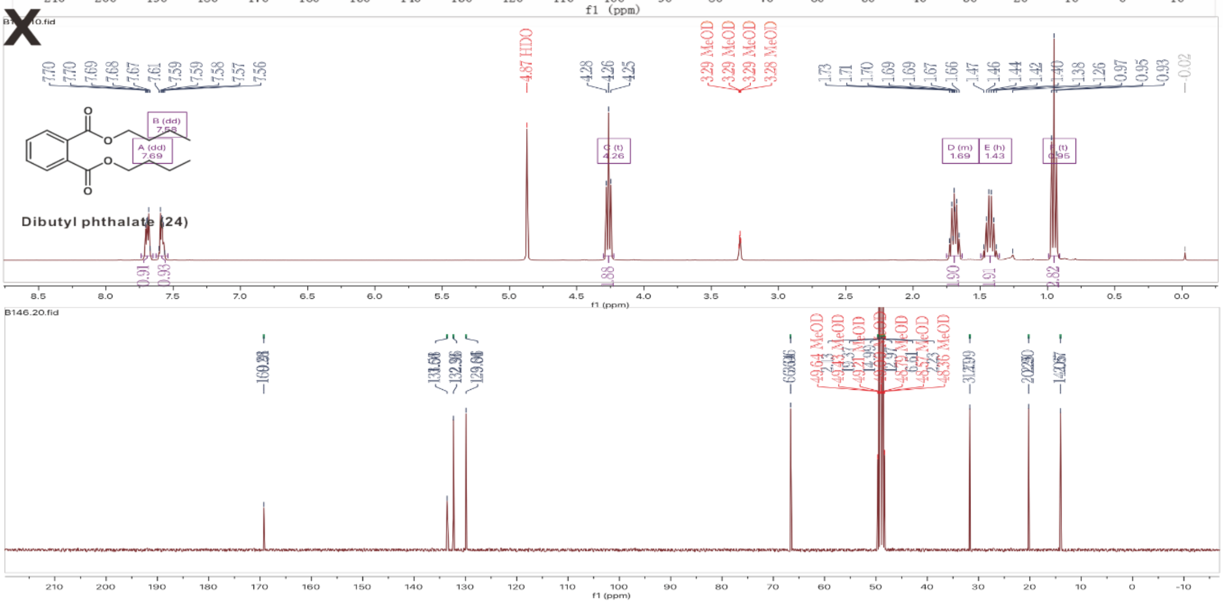
Dibutyl phthalate (24)
B146.20.fid
f1 (ppm)

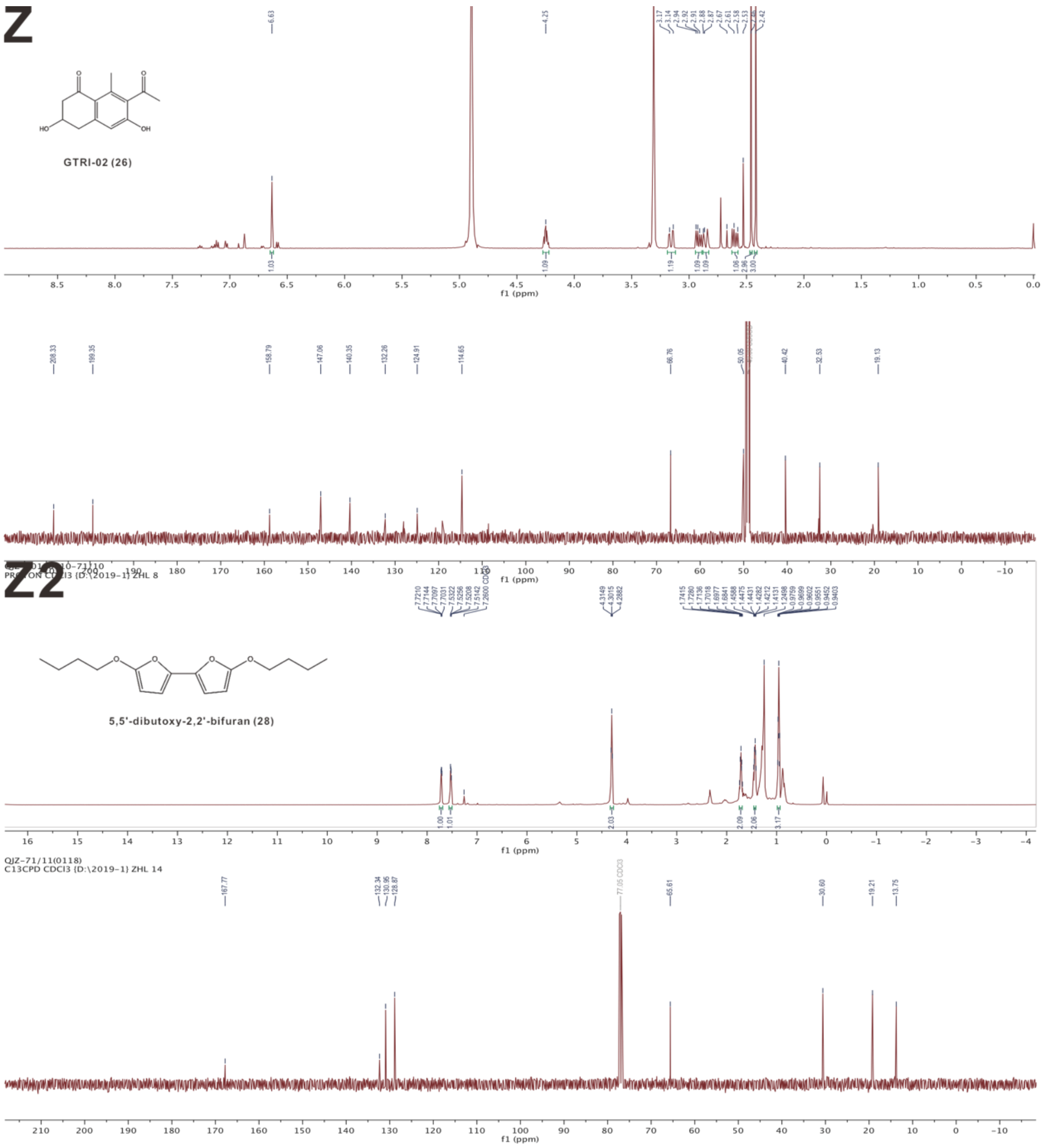

Figure S9. The NMR spectrums of isolates from the strain QHH-9511.

500 MHz for ^{1}H-NMR and 125 MHz for ^{13}C-NMR.

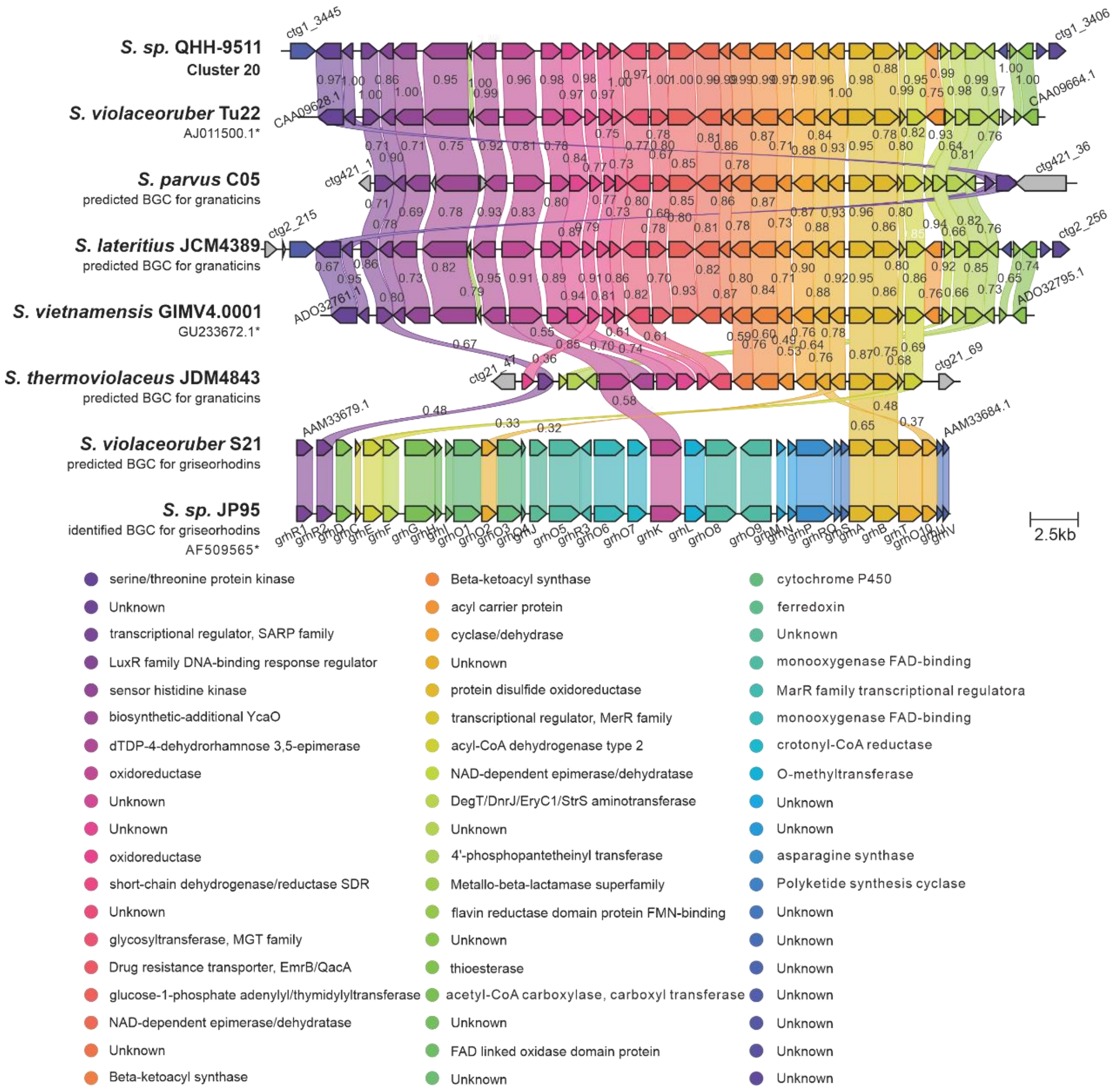

Figure S10. Similarity comparison of identified, and unidentified granaticins BGCs, as well as related BGCs.

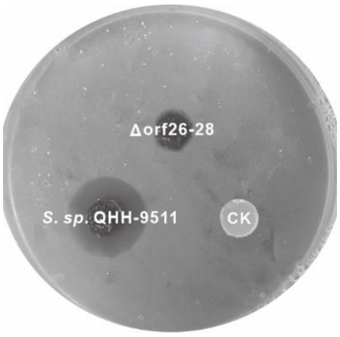

Figure S11. Antimicrobial assay of QHH-9511 and the mutate Δ*orf26-28*.

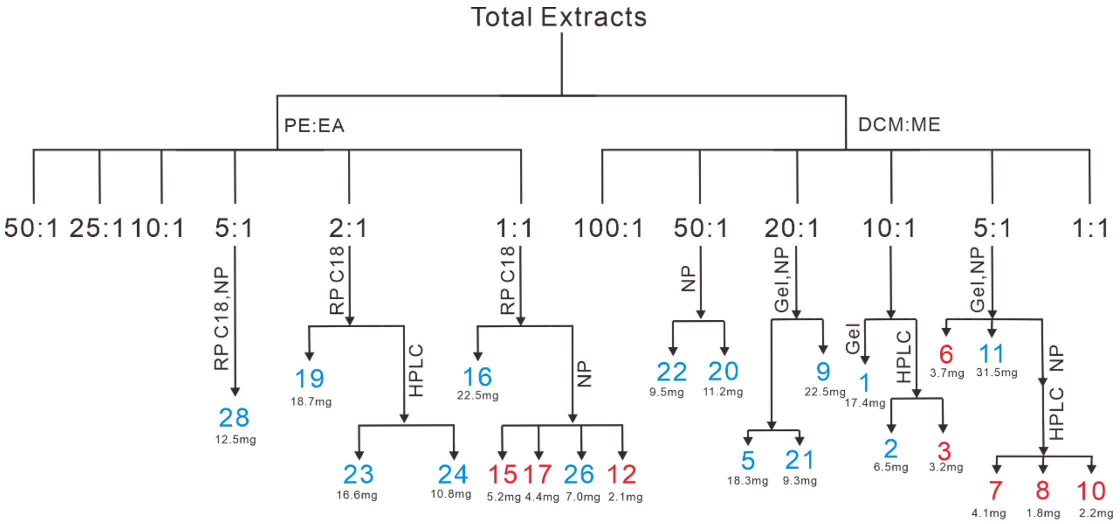

Figure S12. Schematic diagram of the compound separation process.

PE, petroleum ether. EA, ethyl acetate. DCM, dichloromethane. ME, methanol. NP, normal phase. RP C18, reverse phase C18. Gel, Sephadex LH20 gel. HPLC, high performance liquid chromatography.

Table S1| General features of the chromosome of the strain QHH-9511.

Attribute	Chromosome	Plasmid
Genome size (bp)	7524079	91197
GC content (%)	71.34	69.42
Coding protein genes	7555	128
tRNA genes	64	-
rRNA operons	18	-
Genes (total)	7637	128

Table S2| Genomic islands (GIs) distributed on the genome of the strain QHH-9511.

Location	GI No.	Island Start	Island End	Length (bp)	Method	CDS-involved No.
Chr	GI01	1112	24297	23185	A	18
Chr	GI02	34641	57691	23050	A	30
Chr	GI13	65637	98837	33200	A	46
Chr	GI04	106255	122040	15785	A	26
Chr	GI05	129841	135095	5254	A	8
Chr	GI06	266781	274760	7979	A	15
Chr	GI07	313770	322881	9111	A	14
Chr	GI08	590956	605028	14072	A	13
Chr	GI09	816785	865443	48658	A	48
**Chr**	**GI10**	**2148351**	**2293125**	**144774**	**B**	**145**
Chr	GI11	3408106	3420549	12443	A	9
Chr	GI12	3915290	3932190	16900	B	16
Chr	GI13	4432471	4444946	12475	A	19
Chr	GI14	4434769	4451872	17103	B	23
Chr	GI15	4463941	4489992	26051	A	26
Chr	GI16	5163386	5196874	33488	A	29
Chr	GI17	5165254	5197639	32385	B	27
Chr	GI18	6530905	6549920	19015	A	25
Chr	GI19	7209493	7275807	66314	A	62
Chr	GI20	7301455	7324179	22724	A	26
**Chr**	**GI21**	**7342056**	**7470838**	**128782**	**A**	**161**
Chr	GI22	7489833	7508521	18688	A	28

A, IslandPath-DIMOB (https://www.brinkman.mbb.sfu.ca/~mlangill/islandpath_dimob/about.php).

B, Island finder (https://bahamas.triptuner.com/islandfinder/en).

Table S3| BGC Proximity (A) and Resistance models (B) in ARTS2.0 analysis of the strain QHH-9511.

(A)

#Cluster	Type	Source	Location	Core hits	Other hits
cluster-1_1	thiopeptide,LAP	scaffold_1	41013 - 73109	0	2
cluster-1_2	NRPS	scaffold_1	211266 - 272882	0	3
cluster-1_3	T3PKS	scaffold_1	369256 - 408542	0	4
cluster-1_4	NRPS-like	scaffold_1	412513 - 453801	2	3
cluster-1_5	terpene	scaffold_1	488978 - 510344	0	2
cluster-1_6	indole	scaffold_1	631987 - 653286	0	2
cluster-1_7	ectoine	scaffold_1	1739485 - 1749884	0	0
cluster-1_8	butyrolactone	scaffold_1	2649168 - 2658735	0	2
cluster-1_9	siderophore	scaffold_1	2724348 - 2735300	1	0
cluster-1_10	T2PKS,butyrolactone	scaffold_1	3196271 - 3267701	0	4
cluster-1_11	T2PKS	scaffold_1	3733234 - 3805735	6	9
cluster-1_12	melanin	scaffold_1	4775728 - 4786145	0	1
cluster-1_13	ladderane,NRPS	scaffold_1	4842638 - 4894497	3	4
cluster-1_14	other	scaffold_1	5284193 - 5325336	1	5
cluster-1_15	PKS-like	scaffold_1	5334397 - 5375426	5	1
cluster-1_16	T3PKS	scaffold_1	5585816 - 5626950	6	8
cluster-1_17	siderophore	scaffold_1	5703019 - 5717593	2	0
cluster-1_18	terpene	scaffold_1	6400942 - 6426745	0	1
cluster-1_19	NRPS	scaffold_1	6624103 - 6690623	1	3
cluster-1_20	terpene	scaffold_1	6790977 - 6812015	0	1
cluster-1_21	lanthipeptide	scaffold_1	7268274 - 7291028	0	2

(B)

#Model	Description	Sequence id	E-value	Bitscore
RF0007	ABC_efflux	2181	1.50E-97	326
RF0007	ABC_efflux	2195	2.40E-92	308.8
RF0007	ABC_efflux	2459	1.50E-84	283.1
RF0007	ABC_efflux	953	7.00E-80	267.7
RF0051	Chlor_Efflux_Pump	3834	2.60E-111	371.2
RF0053	ClassA	382	2.00E-93	311.1
RF0053	ClassA	2801	7.80E-14	50
RF0054	ClassB	829	2.30E-27	94.5

RF0123	SubclassB1	3412	1.60E-19	68.7
RF0123	SubclassB1	1404	6.10E-15	53.7
RF0155	vanS	271	3.20E-86	288.3
PF00044.19	Gp_dh_N	1667	1.20E-60	202.7
PF00044.19	Gp_dh_N	679	3.30E-39	133.1
PF00185.19	OTCace	5324	7.20E-47	158
PF00185.19	OTCace	1194	1.70E-37	127.6
PF00204.20	DNA_gyraseB	3449	5.10E-62	207
PF00204.20	DNA_gyraseB	5230	2.80E-51	172
PF00227.21	Proteasome	1349	5.30E-38	129.1
PF00227.21	Proteasome	1348	5.80E-27	93.1
PF00364.17	Biotin_lipoyl	1933	3.60E-45	150.3
PF00364.17	Biotin_lipoyl	3353	1.30E-23	81.3
PF00364.17	Biotin_lipoyl	2480	2.20E-18	64.5
PF00364.17	Biotin_lipoyl	3369	2.60E-17	61.1
PF00364.17	Biotin_lipoyl	2915	3.70E-17	60.6
PF00364.17	Biotin_lipoyl	4327	3.70E-17	60.6
PF00364.17	Biotin_lipoyl	3892	2.10E-16	58.2
PF00364.17	Biotin_lipoyl	5889	3.10E-06	25.6
PF00521.15	DNA_topoisoIV	3448	8.00E-149	494.9
PF00521.15	DNA_topoisoIV	5236	6.50E-145	482.1
PF01039.17	Carboxyl_trans	4983	4.10E-201	667.4
PF01039.17	Carboxyl_trans	4334	1.90E-200	665.3
PF01039.17	Carboxyl_trans	2903	2.00E-191	635.5
PF01039.17	Carboxyl_trans	2479	2.10E-163	543.1
PF01039.17	Carboxyl_trans	3891	1.20E-139	464.7
PF01039.17	Carboxyl_trans	2173	7.40E-25	86.1
PF13599.1	Pentapeptide_4	5864	3.10E-23	80.3
PF13599.1	Pentapeptide_4	6671	2.80E-14	51.5
PF13599.1	Pentapeptide_4	4053	3.60E-14	51.2
PF13599.1	Pentapeptide_4	3791	1.50E-12	46
TIGR02013	TIGR02013	4077	0	1815.2
TIGR00663	TIGR00663	3453	1.20E-90	302.7
TIGR00663	TIGR00663	4975	1.80E-64	216.6

Table S4| The NMR data of several aromaticpolyketides.

Compounds	^{1}H NMR δ	^{13}C NMR δ	Reference	Yield (mg/L)
**Granaticinic acid (1)**	(500 MHz, ACE-d$_6$) 5.45 (1H), 5.12 (2H,H15,H17), 4.92 (1H), 4.68(1H,H4), 4.32(1H,H3), 3.96 (H,H19), 3.73 (1H,H21), 3.22 (1H), 2.68 (3H,H2,H18b), 1.58 (3H,H16), 1.49 (1H,H18a), 0.95 (3H,H22).	(126 MHz, ACE-d$_6$) 17.1(C16), 18.2(C22), 37.3(C2), 37.4(C18), 62.5(C4), 61.8(C17), 67.4(C3), 67.5(C15), 69.7C19), 71.8(C21), 73.2, 81.2(C20), 111.3(C12), 112.5(C7), 132.8(C14), 140.0(C10), 144.6(C9), 146.9(C5), 175.5(C13)	(3)	4.35
**6-deoxy-13-hydroxy-8,11-dione-dihydrogranaticins A (2)**	(500 MHz, ACE-d$_6$) 7.43(1H,H6), 5.11(1H,H15), 4.95(1H,H17), 4.43(1H,H3), 3.94(1H,H19), 3.69(1H,H21), 2.98(1H,H4b), 2.76(1H,H4b), 2.65(2H,H2a,H18a), 2.57(1H,H2b), 1.56(3H,H16), 1.50(1H,H18b), 1.03(3H,H22).	(126 MHz, ACE-d$_6$) 192.43(C11), 179.72(C8), 172.11(C1), 158.77(C13), 147.43(C9), 144.64(C5), 140.85(C10), 135.90(C14), 130.68(C12), 120.96(C6), 113.51(C7), 80.96(C20), 73.36(C21), 72.13(C19), 68.60(C15), 64.33(C3), 62.71(C17), 40.87(C2), 37.28(C18), 34.75(C4), 19.06(C16), 17.16(C22).	(4)	1.63
**Diglycosyl-MM44785(5)**	(500 MHz, MeOD) 5.16 (H,H17),5.01(H15) 4.41 (1H,H3), 3.92 (1H,H19), 3.73 (1H,H21), 2.94 (1H, H2a), 2.83 (1H, H2b), 2.68(1H,H18a),2.66 (1H,H4a), 2.44 (1H), 2.26 (1H,H4b), 1.46(H18b),1.35 (3H,H16), 0.85 (3H,H22).	(126 MHz, MeOD) 200.33(C13), 195.63(C6), 169.25(C1), 154.88(C11), 150.01(C8), 138.30(C9), 135.50(C10), 113.02(C7,C12), 84.75(C5), 81.64(C20), 76.53(C14), 73.57(C21), 72.07(C19), 66.84(C15), 65.98(C3), 63.59(C17), 38.19(C18), 36.09(C2), 24.27(C4), 17.03(C22), 15.98(C16).	(5)	4.57
**Granaticin A (9)**	(500 MHz, ACE-d$_6$) 13.10(1H,8-OH), 12.86(1H,11-OH), 5.45(1H, H4), 5.15(1H,H5), 5.11(1H,H17), 4.92(1H,H3), 3.98(1H,H19), 3.73(1H,H21), 3.50(1H,H4'), 3.22(1H,H2a), 2.59(1H,H18a), 2.48(1H,H2b), 1.59(3H,H16), 1.51(1H, H18b), 0.95(3H,H22).	(126 MHz, MeOD) 177.42(C6), 174.05(C13), 172.71(C1), 171.43(C11), 167.72(C8), 147.79(C5), 144.48(C9), 139.57(C10), 133.02(C14), 113.10(C7), 111.91(C12), 81.46(C20), 73.85(C21), 72.31(C19), 70.86(C15), 68.21(C3), 67.88(C4), 63.18(C17), 37.77(C2), 37.68(C18), 18.34(C16), 17.10(C22).	(6)	5.63
**Granaticin B (11)**	(500 MHz, ACE-d$_6$) 12.94(8-OH), 12.86(11-OH), 5.45(1H,H4), 5.16(2H,H5,H17), 4.92(1H,H3), 4.83(1H,H1'), 4.09(1H,H5'), 4.06(1H,H19), 3.80(1H,H21), 3.46(1H,H4'), 3.21(1H,H2a), 2.68(1H,H18a), 2.51(1H,H2b), 1.96(1H,H3'a), 1.81(1H,H3'b), 1.62(1H,H16), 1.60(1H,H2'a), 1.28(1H,H2'b), 1.20(1H,H6'), 0.95(3H,H22).	(126 MHz, ACE-d$_6$) 175.9(C6), 175.3(C13), 172.9(C1), 172.1(C11), 147.6(C5), 144.1(C9), 140.2(C10), 133.1(C14), 112.5(C7), 111.3(C12), 96.4(C1'), 79.4(C20), 77.3(C5'), 73.3(C21), 69.6(C19), 68.2(C4), 67.5(C4'), 67.5(C17), 66.9(C3), 62.4(C15), 41.2, 37.4(C2), 35.9(C18), 26.2(C2'), 24.5(C3'), 18.4(C16), 17.7(C6'), 17.2(C22).	(7)	7.88
**aloesaponarin II (16)**	(500 MHz, DMSO-d$_6$) 12.84 (1H, 1-OH), 7.50 (1H,H4), 7.42 (1H,H2), 7.23 (1H,H5), 7.11 (1H,H3), 6.81 (1H,H7), 2.30 (3H, 8-CH$_3$).	(126 MHz, DMSO-d$_6$) 189.15(C9), 182.40(C10), 163.15(C1), 161.48(C6), 145.38(C8a), 136.93(C5a), 135.86(C3), 132.62(C4a), 125.3(C7), 124.74(C3), 124.22(C8), 118.20(C4), 116.55(C2), 112.36(C5), 23.60(C8-CH$_3$).	(8)	5.63
**BSM2 (19)**	(500 MHz, MeOD) 7.65(1H,H5), 7.49(1H,H6), 7.16(1H,H4), 6.12(1H,H10), 4.29(2H,H2), 3.40(3H,1-OCH$_3$), 2.32(3H,H12).	(126 MHz, MeOD) 208.47(C9), 181.79(C1), 168.18(C11), 159.41(C7), 137.62(C3), 134.43(C5), 130.23(C4), 122.28(C8), 118.68(C6), 111.64(C10), 50.38(1-OCH$_3$), 29.98(C2), 20.07(C12).	(9)	4.68
**BSM1 (20)**	(500 MHz, MeOD) 11.8(1H,H1), 7.65(1H,H5), 7.49(2H,H6), 7.23(3H,H4), 6.14(1H,H10), 4.18(2H,H2), 2.40(3H,H12).	(126 MHz, MeOD) 182.04(C9), 175.30(C1), 168.04(C11), 159.36(C7), 137.36(C3), 134.39(C5), 130.00(C4), 122.69(C8), 118.83(C6), 111.73(C10), 41.63(C2), 20.04(C12).	(9)	2.80
**Fogacin (21)**	(500 MHz, MeOD) 7.70(1H,H6), 5.19(1H,H15), 4.92(1H,H8), 4.45(1H,H3), 3.49(1H,H15), 3.08(1H,H10b), 2.89(1H,H4b), 2.69(1H,H10a), 2.56(1H,H2), 2.46(1H,H9b), 2.10(1H,H9a), 1.48(3H,H16).	(126 MHz, MeOD) 205.70(C11), 172.48(C1), 159.03(C13), 146.41(C7), 143.48(C5), 127.07(C14), 118.64(C6), 113.84(C12), 68.48(C15), 67.52(C8), 64.41(C3), 41.15(C2), 35.89(C10), 34.96(C4), 32.59(C9), 19.16(C16).	(10)	2.33
**Phaeochromycin I (22)**	(500 MHz, MeOD) 7.63 (1H,H7), 7.44 (1H,H8), 7.12 (1H,H6), 6.09 (1H,H3), 4.31 (1H,H1'), 4.19 (1H,H4'), 2.91	(126 MHz, MeOD) 208.74(C2'), 181.84(C4), 175.49(C6'), 168.43(C2), 159.20(C8a), 136.90(C5),	(9)	2.38

	(2H), 2.57 (1H,H3'), 2.47 (1H), 2.35 (3H,H9), 2.16(1H5'b), 1.96(1H,H5'a).	134.56(C7), 130.35(C6), 121.92(C4a), 118.71(C8), 111.47(C3), 65.60(C4'), 50.41(C3'), 50.16(C1'), 42.61(C5'), 20.15(C9).		
**n-butyl isobutyl terephthalate (23)**	(500 MHz, $CDCl_3$) 7.72 (2H,H3,H6), 7.53 (2H,H1,H5), 4.30 (2H,H9), 4.08 (2H,H9'), 2.04 (1H,H10), 1.72 (2H,H10'), 1.43 (2H,H11'), 0.99 (6H,H11), 0.96 (3H,H12').	(126 MHz, $CDCl_3$) 167.84(C7), 167.81(C7'), 132.54(C1), 132.48(C4), 131.05(C2,C5), 128.99(C3,C6), 71.93(C9), 65.70(C9'), 30.72(C10), 27.87(C9'), 19.33(C10'), 19.30(C11'), 13.86(C11a,C11b).	(11)	4.15
**dibutyl phthalate (24)**	(400 MHz, MeOD,) 7.69 (2H, H2, H2'), 7.58 (2H, H1, H1'), 4.26 (4H,H6,H6'), 1.69 (4H,H7,H7'), 1.43 (4H,H8,H8'), 0.95 (6H,H9,H9').	(101 MHz, MeOD) 169.28(C4,C4'), 133.58(C2,C2'), 132.33(C1,C1'), 129.86(C3,C3'), 66.64(C6,C6'), 31.71(C7,C7'), 20.25(C8,C8'), 14.05(C9,C9')	(12)	2.70
**GTRI-02 (26)**	(500 MHz, MeOD) 6.65(1H,H5), 4.27(1H,H3), 2.96-2.85(2H,H4a,H4b), 2.63(2H,H2a H2b), 2.48(3H, H10), 2.44(3H,H11).	(126 MHz, MeOD) 208.31(C9), 199.32(C1), 158.78(C6), 147.05(C4a), 140.35(C8), 132.24(C7), 124.90(C8a), 114.65(C5), 66.75(C3), 50.03(C2), 40.40(C4), 32.54(C10), 19.14(C11).	(13)	1.75
**5,5'-dibutoxy-2,2'-bifuran (28)**	(500 MHz, $CDCl_3$) 7.71 (2H,H3,H3'), 7.52 (2H,H4,H4'), 4.30 (4H,H6,H6'), 1.71 (4H,H7,H7'), 1.43 (4H,H8,H8'), 0.96 (6H,H9,H9').	(126 MHz, $CDCl_3$) 173.5(C5,C5'),167.77(C2,C2'), 132.34, 130.95(C4,C4'), 128.87(C3,C3'), 65.61(C6,C6'), 30.60(C7,C7'), 19.21(C8,C8'), 13.75(C9,C9').	(14)	3.13

Table S5| The minimum inhibitory concentration test for seven compounds using human pathogen and phytopathogen.

**Indicator strain**	GTRI-2 (**29**)	BSM1 (**23**)	Fogacin (**24**)	6-Deoxy-13-hydroxy-8,11-dione-dihydrogranaticin A (**2**)	BSM2 (**22**)	Granaticin A (**9**)	Granaticin B (**11**)
Staphylococcus aureus ATCC 43300 (methicillin-resistant) (human pathogen)	>256 [a]	>256	>256	4	>256	0.5	1
Staphylococcus aureus ATCC 29213 (methicillin-sensitive) (human pathogen)	>256	>256	>256	4	>256	0.5	1
Bacillus cereus IOP 01 (human pathogen)	>256	>256	128	8	>256	0.25	0.125
Erwinia carotovora pv. *Carotovora* NPCB 01 (cabbage soft rot)	>256	>256	256	16	>256	0.5	0.125
Erwinia carotovora subsp. *Carotovora* (Jone) NPCB A3 (amorphophallus soft rot)	>256	>256	256	4	>256	2	1
Pseudomonas syringae pv. *Actinidiae* NPCB 201 (kiwifruit canker pathogen)	>256	>256	128	4	>256	2	0.5
Ralstonia solanacearum NPCB 002 (night shade plant pathogens)	>256	>256	256	32	>256	2	0.5

[a]minimum inhibitory concentration (μg/mL)

Table S6| Deduced functions of the open reading frames of the putative granaticins cluster in the strain QHH-9511.

Protein	Amino acid	Proposed function	Seqence similarty (protein, origin)	Identity (%)
Orf1	434	Serine/threonine-protein kinase	WP_030930457 *Streptomyces sp.* NRRL B-24720	75
Orf2	182	transcriptional regulator	WP_028797751 *Streptomyces purpureus*	97
Orf3	186	AfsR/SARP family transcriptional regulator	WP_137988230.1 *Streptomyces exfoliatus*	90
Orf4	154	putative two-component response regulator	CAA09631.1 *Streptomyces violaceoruber*	81
Orf5	406	hypothetical protein	WP_137988232.1 *Streptomyces exfoliatus*	82
Orf6	773	TOMM precursor leader peptide-binding protein	WP_137988233 *Streptomyces exfoliatus*	84
Orf7	57	hypothetical protein	ADO32767 *Streptomyces vietnamensis*	77
Orf8	434	DUF1205 domain-containing protein	WP_137988234 *Streptomyces exfoliatus*	98
Orf9	531	MFS transporter	WP_137988235 *Streptomyces exfoliatus*	94
Orf10	353	dTDP-1-glucose synthase	AAA99940 *Streptomyces violaceoruber*	96
Orf11	83	dTDP-glucose 4,6-dehydratase	WP_137994572.1 *Streptomyces exfoliatus*	98
Orf12	202	putative cyclase-dehydratase	CAA09639.1 *Streptomyces violaceoruber*	98
Orf13	130	DsbA family oxidoreductase	WP_052499203 *Streptomyces vietnamensis*	81
Orf14	196	putative transcriptional activator	ADO32774.1 *Streptomyces vietnamensis*	84
Orf15	400	hydroxylase	AJF69847 *Streptomyces vietnamensis*	88
Orf16	304	dTDP4-keto-6-deoxyhexose reductase	WP_065482219 *Streptomyces sp.* PTY087I2	80
Orf17	433	dTDP-2,6-dideoxy-4-ketoglucose 3-dehydrase	WP_041130153 *Streptomyces vietnamensis*	93
Orf18	410	hypothetical protein	CAA09645 *Streptomyces violaceoruber*	85
Orf19	195	dTDP-4-keto-6-deoxy-D-glucose epimerase	WP_065482211 *Streptomyces sp.* PTY087I2	91
Orf20	342	dTDP-3,4-diketo-2,6-dideoxyglucose ketoreductase	Q9ZA33 *Streptomyces violaceoruber*	99
Orf21	443	dTDP-4-keto-6-deoxyglucose-3,5-epimerase	ADO32777 *Streptomyces vietnamensis*	93
Orf22	266	hypothetical protein	WP_109197474 Streptomyces sp. CS014	76
Orf23	351	LLM class flavin-dependent oxidoreductase	WP_041130149 *Streptomyces vietnamensis*	93
Orf24	249	polyketide ketoacyl reductase （KR）	actIII *Streptomyces coelicolor* A3(2)	99
Orf25	220	polyketide keto-reductase for C9 （KR）	ADO32785 *Streptomyces vietnamensis*	92
Orf26	421	Polyketide alpha-Ketoacyl synthase（KS_{α}）	actIOrf1 *Streptomyces coelicolor* A3(2)	98
Orf27	412	Polyketide ketoacyl synthase chain-length factor (CLF, KS_{β})	actIOrf2 *Streptomyces vietnamensis*	65
Orf28	85	acyl carrier protein (ACP)	actIOrf3 *Streptomyces coelicolor* A3(2)	82
Orf29	323	Aromatase (ARO) (First ring aromatization)	ADO32789 *Streptomyces violaceoruber*	95
Orf30	213	hypothetical protein	ADO32790 *Streptomyces vietnamensis*	52
Orf31	142	nuclear transport factor 2 family protein	WP_065482178 *Streptomyces sp.* PTY087I2	93
Orf32	277	4'-phosphopantetheinyl transferase superfamily protein	WP_079137757 *Streptomyces sp.* PTY087I2	67
Orf33	321	cyclase	ADO32793 *Streptomyces vietnamensis*	90
Orf34	184	flavin reductase family protein	WP_065482172 *Streptomyces sp.* PTY087I2	81
Orf35	161	Uncharacterized protein	ADL30_11960 *Streptomyces sp.* NRRL S-1521	37
Orf36	124	hypothetical protein	WP_137988261 *Streptomyces exfoliatus*	82
Orf37	257	transcriptional regulator	WP_147979386 *Streptomyces sp.* ms191	77

3. References

1. Kieser T, Bibb MJ, Buttner MJ, Chater KF, Hopwood DA. 2000. Practical *Streptomyces* Genetics: A Laboratory Manual Norwich, U.K., The John Innes Foundation.
2. Macneil DJ, Gewain KM, Ruby CL, Dezeny G, Macneil T. 1992. Analysis of *Streptomyces avermitilis* genes required for avermectin biosynthesis utilizing a novel integration vector. Gene 111:61-68.
3. Maehr H, Cuellar HV, Smallheer J, Williams TH, Sasso GJ, Berger J. 1979. Microbial products. II. Granaticinic acid, a new antibiotic from a thermophilic streptomycete. Monatshefte für Chemie / Chemical Monthly 110:531-540.
4. Jiang B, Li S, Zhao W, Li T, Zuo L, Nan Y, Wu L, Liu H, Yu L, Shan G, Zuo L. 2014. 6-Deoxy-13-hydroxy-8,11-dione-dihydrogranaticin B, an intermediate in granaticin biosynthesis, from *Streptomyces* sp. CPCC 200532. J Nat Prod 77:2130-3.
5. Deng M, Zhu C, Guo J, Zhu H. 2011. Isolation and Identification of Violet Blue Pigment in *Streptomyces vietnamensis*. Journal of South China University of Technology ((Natural Science Edition) 39:132-137.
6. Chang C-J, Floss HG, Soong P, Chang C-T. 1975. Identity of the antitumor antibiotic litmomycin with granaticin A. The Journal of antibiotics 28:156-156.
7. Gilpin ML, Box SJ, Elson AL. 1988. New quinone antibiotics of the granaticin type, isolated from *Streptomyces lateritius*. II. Structure determination. J Antibiot (Tokyo) 41:512-8.
8. Cui HX, Shaaban KA, Schiebel M, Qin S, Laatsch HJWJoM, Biotechnology. 2008. New antibiotic with typical plant anthraquinone structure obtained studying terrestrial and marine Streptomycetes. 24:419-421.
9. Kalaitzis JA, Moore BS. 2004. Heterologous Biosynthesis of Truncated Hexaketides Derived from the Actinorhodin Polyketide Synthase. Journal of Natural Products 67:1419-1422.
10. Radzom M, Zeeck A, Antal N, Fiedler H-P. 2006. Fogacin, a Novel Cyclic Octaketide Produced by *Streptomyces* Strain Tü 6319 †. The Journal Of Antibiotics 59:315.
11. Sun YL, Chen Y, Xin-Ya X, Zhang XY, Zheng ZH, Nong XH, Bao J, Shu-Hua J. 2013. Secondary Metabolites of Marine-derived Fungus *Penicillium oxalicum* SCSGAF 0023 and Their Antifouling and Enzyme-inhibitory Activities. Natural Product Research Development.
12. Dai HF, Mei WL, Peter P, Lin WH. 2006. Studies on the tumor cytotoxic constituents from the marine sponge *Hyrtios erectus*. Chinese Journal of Marine Drugs.25:1-5
13. Hyung YEOW-, Sik YUNB-, Seock KIMS-, Kyung PE-, Ho KIMY-, Dong YOOI-, Hun YUS-. 1998. GTRI-02, a New Lipid Peroxidation Inhibitor from *Micromonospora* sp. SA246. J Antibiot 51:952-953.
14. Jie L, Jing X, Xiao JZ, Wen YG, Shu ZZ, Yuan QG. 2010. A new heterocyclic compound from *Cyathula officinalis* Kuan. Chinese Chemical Letters 21:70-72.
